# Trends in assisted dying among patients with psychiatric disorders and dementia in Belgium: A health registry study

**DOI:** 10.1371/journal.pmed.1004522

**Published:** 2025-11-19

**Authors:** Jacques Wels, Natasia Hamarat

**Affiliations:** 1 University College London, Unit for Lifelong Health and Ageing (LHA), London, United Kingdom; 2 Université libre de Bruxelles, Health & Society Research Unit, Brussels, Belgium; 3 Université libre de Bruxelles, Centre de droit public et social, Brussels, Belgium; University of Cambridge, UNITED KINGDOM OF GREAT BRITAIN AND NORTHERN IRELAND

## Abstract

**Background:**

Assisted dying and euthanasia (ADE) for patients with psychiatric disorders or dementia have increased in jurisdictions where the practice is legal. In this study, we examine trends in euthanasia cases involving patients with these conditions in Belgium, where the law makes a distinction based on whether a patient’s death is not expected in the foreseeable future (>12 months)—a common situation in cases of dementia or psychiatric disorders.

**Methods and findings:**

We use data on all cases of euthanasia reported to the Federal Commission for the Control and Evaluation of Euthanasia from 2002 (when the legislation was introduced) to 2023 (*N* = 33,592). Psychiatric disorders and dementia represent 1.27% and 0.92% of all cases, respectively. Using time-series zero-inflated negative binomial regression, we model trends by first examining interactions between euthanasia reasons and year, then extending to three-way interactions with patients’ characteristics. The model calculates change in count and is replicated with an offset to account for demographic changes and generate rates. Our results show that euthanasia for psychiatric disorders and dementia showed distinct trends over time. Although slightly increasing, euthanasia for psychiatric disorders followed trends similar to the other types of euthanasia (count = 1.00 [95%CI: 0.98; 1.03]—rate = 1.02 [95%CI: 0.99; 1.04]), while euthanasia cases for dementia increased faster than other types of euthanasia (count = 1.03 [95%CI: 1.00; 1.06]—rate = 1.04 [95%CI: 1.01;1.07]). Trends in euthanasia for dementia and psychiatric disorders coincide with demographic changes. While euthanasia rates for psychiatric disorders were initially higher among women, the rate among men has been increasing over time. Regional trends show higher overall euthanasia rates in the Dutch-speaking population, but with faster increases in the French-speaking population. A key limitation of this study is the lack of information on patients’ socio-economic profiles.

**Conclusions:**

In Belgium, between 2002 and 2023, there are distinct trends for euthanasia for non-terminal illnesses. Euthanasia for psychiatric disorders followed similar trends as euthanasia for terminal illnesses, whereas euthanasia cases involving cognitive conditions increased at a faster rate. Furthermore, there were gender and regional differences, which diminished over time.

## Author summary

**Why was this study done?:**

- Assisted dying and euthanasia (ADE) for people with psychiatric disorders or dementia often raise ethical concerns, especially in countries considering legalisation.
- We studied how often euthanasia is performed for these conditions in Belgium, where it has been legal since 2002.

**What did the researchers do and find?:**

- We analysed all reported euthanasia cases in Belgium from 2002 to 2023, focussing on those involving psychiatric disorders or dementia.
- These cases made up a small portion of all euthanasia cases—less than 2.5% combined. We found that euthanasia for psychiatric disorders has increased at a similar rate to all euthanasia cases, whereas cases of euthanasia for dementia have increased slightly more. There were gender and regional differences in these trends

**What do these findings mean?:**

- Increase of cases of euthanasia for psychiatric disorders have increased in line with the total increase in cases for all other reasons while cases for dementia have increased more than other cases.
- A major limitation of the study is the lack of data on patients’ socio-economic status.

## Abstract 3

In this registry study, Jacques Wels and Natasia Hamarat use data on all cases of euthanasia in Belgium between 2002-2023 to assess trends in assisted dying for patients with psychiatric disorders and dementia.

## Background

A central point of contention in the debate surrounding the implementation or expansion of assisted dying and euthanasia (ADE) regulations concerns the permissibility of such practices for individuals with non-terminal illnesses. The discourse primarily focuses on two categories of conditions where death is not expected in the foreseeable future: dementia and other degenerative disorders, and severe psychiatric illnesses.

Empirical research on ADE using data is sparse [1], and much of the scientific literature—often consisting of opinion pieces [2,3]—relies on ethical debates [4]. When data are mobilised, they are often examined at a very descriptive level, focussing on ADE trends while overlooking changes in population structures [4,5]. We have recently demonstrated that failing to account for population change when analysing annual ADE cases can lead to trends that are overstated due to demographic shifts. For example, population ageing contributes to increased number of cases, as euthanasia rates are higher among older age groups [4].

The observed increase in cases fuel concerns about a gradual expansion of eligibility criteria, particularly leading to concerns regarding vulnerable patients [5–9]. These concerns are used as an argument against implementing ADE for non-terminal illness in other jurisdictions and as an argument in favour of introducing stricter safeguards [8–13].

The idea that eligibility criteria in ADE regulations are gradually expanded, or that vulnerable populations are at greater risk is not substantiated by empirical studies [14–16]. This does not mean that sub-groups differences in ADE do not exist and that trends are not evolving over time. Many studies have focussed on gender and regional discrepancies. For instance, it was shown that female patients are notably overrepresented in psychiatric cases, although these comprise a small fraction of total cases [17,18]. Gender disparities have been noted in the context of Belgian euthanasia data, which shows a relatively balanced distribution with females representing 49.6% of euthanasia cases in 2020 [19] and data on euthanasia as the ratio on all deaths by gender show similar rates among genders [20]. Regional differences have also been documented. For example, in the Netherlands, unexplained geographical variations in euthanasia incidence were observed across provinces. Factors such as age, church attendance, political orientation, income, self-perceived health, and availability of voluntary workers have been associated with these differences, yet a significant portion of the variation remains unexplained [21]. In Belgium, official statistics reveal higher propensities for euthanasia in the Flemish region [22], with most research predominantly focussed on Flanders [23,24].

Using administrative data on all euthanasia cases reported to the Belgian Federal Commission for the Control and Evaluation of Euthanasia (FCCEE) between 2002 and 2023, this study examines trends in the practice of euthanasia by addressing two research questions: (R.Q.1) How do trends in euthanasia cases involving psychiatric conditions and cognitive disorders compare with those for other reasons, such as terminal illness, and how does this comparison change when demographic composition is taken into account?; (R.Q.2) Are distinct patterns observable across population sub-groups, such as gender and region?

## Data and methods

### Ethics statement

The data used in this study are fully anonymized and encompass all reported euthanasia cases since 2002. the Belgian FCCEE granted ethical approval on May 14, 2024. To obtain approval, we submitted a detailed research proposal to the FCCEE outlining the study objectives, methodology, and data protection measures. The Commission reviewed and approved the project, authorising access to anonymized data. This is done in full compliance with the 2002 Belgian law governing euthanasia and data protection. Consent to participate was waived by the FCCEE in accordance with Belgian regulations.

### Data

We use data routinely collected by the FCCEE, derived from individual reports submitted by euthanasia practitioners. These reports are fully anonymized and encompass all reported euthanasia cases since 2002, including information on the reasons for euthanasia, as well as the patients’ gender, age group, and language. The dataset includes 33,647 cases, representing all reported euthanasia cases in Belgium between 2002 and 2023. Since the euthanasia law was implemented in mid-2002, we exclude data from 2002 in our empirical models because the law was implemented in mid-2002 leading to low cases (*N* = 24). Additionally, 43 cases were removed due to incomplete information. No imputations were made to address the missing data, given the small proportion (0.1% of the total) and limited available information. The final sample includes 33,623 cases. Among these, 427 cases were justified by psychiatric disorders and 310 cases by dementia, i.e., respectively, 1.27% and 0.92% of all cases of euthanasia observed over the period.

The dataset contains aggregated count data stratified by multiple demographic, temporal, and contextual factors. Each row represents a unique combination of attributes, including year, language, gender, age, age group, location, and reason for the case. The primary variable of interest is the count of cases for each combination of factors, with an additional variable providing the total count of cases aggregated by year. The dataset is structured as a rectangular table, with each row corresponding to a unique combination of predictor variables, ensuring that all possible combinations are represented, including those with zero counts.

### Reasons for euthanasia

Under Belgian law, euthanasia is defined as the “act performed by a third party who intentionally ends a person’s life at that person’s request”. Eligible patients must experience “constant and unbearable physical or mental suffering that cannot be alleviated” resulting from a serious and incurable medical condition. The law imposes strict criteria, including the following: (1) the patient must be legally an adult or an emancipated minor, competent, and conscious when making the request; (2) the request must be voluntary, well-considered, repeated, and free from external pressure; (3) the patient must have a serious and incurable medical condition caused by illness or accident with no prospect of improvement; (4) they must be experiencing constant and unbearable physical or mental suffering that cannot be alleviated; (5) the patient must be fully informed about their condition, prognosis, treatment options, and palliative care; (6) the physician must confirm that all criteria are met and consult the treatment team; (7) an independent doctor must be consulted, and in non-terminal cases, also a psychiatrist or relevant specialist, with a 1-month waiting period; (8) the request must be made in writing by the patient; (9) the physician must submit the euthanasia declaration form to the FCCEE within four working days following the procedure, which retrospectively assesses legal compliance and may refer cases to the public prosecutor if necessary [25].

In other words, in cases of euthanasia when death is not expected in the foreseeable future (defined by the FCCEE as a period of 12 months or more)—such as for psychiatric disorders or dementia or cognitive conditions—a reinforced procedure applies. In this case, a second physician (i.e., independent psychiatrist or specialist in the condition prompting the euthanasia) should be consulted, and a mandatory waiting period applies [25]. Euthanasia is typically administered intravenously, but rare cases of supervised self-administration exist (0.3% of all cases in 2022−2023 [26]).

Since 2014, the scope of the law has extended euthanasia access to non-emancipated minors under strict conditions. The minor must demonstrate the capacity for discernment, be suffering from a physical (not psychiatric) condition that is incurable and leads to death in the near term, and receive the explicit consent of their parents or legal representatives. These cases are rare, with five recorded between 2014 and 2023 [26]. Therefore, they are excluded from this study.

The FCCEE identifies 12 medical conditions that may justify euthanasia (post-coded based on specific conditions mentioned by the medical practitioner on the euthanasia certificate) [27]: cancer and tumours, multimorbidity, nervous system diseases, psychiatric disorders, dementia (cognitive disorders), diseases of the respiratory system, diseases of the circulatory system, diseases of the genitourinary system, diseases of the digestive system, haematological disorders, endocrine, nutritional, and metabolic diseases, musculoskeletal and connective tissue diseases. We maintain the FCCEE’s distinction between psychiatric and cognitive disorders due to their differing clinical profiles. For analytical purposes, we group the reasons for euthanasia into three broad categories: (1) psychiatric disorders, (2) dementia and (3) all other justifications, excluding psychiatric disorders and dementia.

It should be noted that cases involving multimorbidity may include dementia or psychiatric disorders. Among patients who received euthanasia with a multimorbidity profile between 2002 and 2023, 11.3% had psychiatric disorders and 4.9% had dementia or memory disorders. However, with an average number of recorded conditions per patient of between 2.5 and 3, these pathologies co-occurred with other illness(es) and these cases were excluded from this study.

For the purposes of this paper, “assisted dying” serves as an umbrella term for assisted dying, assisted suicide and euthanasia. The term “euthanasia” is used when discussing Belgium, in alignment with its statutory language [28].

### Covariates

The dataset includes several key variables for analysis. The year of euthanasia, recorded from 2002 to 2023, is used as a continuous variable (coded 0–22) in main analyses, with a sensitivity check treating it as categorical to address non-linear trends. Age is categorised into eight groups—15–29, 30–39, 40–49, 50–59 (reference), 60–69, 70–79, 80–89, and 90+—to ensure anonymity. Gender, reported by the medical practitioner, is recorded as male or female (reference). To account for regional differences within Belgium’s federal structure, we include language data (Dutch or French, reference) used by the reporting practitioner, as place of residence was inconsistently collected.

Additional variables type of unbearable suffering (physical, reference; mental; or both), basis for euthanasia (advanced request, made beforehand and valid in cases of irreversible coma, or actual request, which must be reaffirmed by the patient at the time of euthanasia, reference), and place of death, categorised as home (reference), hospital, care home, palliative care, or other. We also include a variable on the term of death (expected within a year (reference) or longer), as the Belgian law distinguishes between death expected in the near term and death that is not, which determines whether a standard or reinforced procedure applies (involving the attending physician and two other physicians independent from each other and from the patient, including a psychiatrist or specialist, and a mandatory 1-month delay between the written request and the act). Only patients with non-evolving or very slowly progressive conditions are considered to fall under the latter [25].

### Population offset

We generate population figures based on demographic data retrieved from Federal Agency for National Statistics (*Statbel*). This data includes information on the total population as of January 1st for each selected year (2002–2023), broken down by age group, sex, and region of residence. We chose to use population figures instead of the number of deaths by sub-group, as done in previous studies [5,21,29,30], because a non-negligible share of euthanasia is performed on patients not expected to die in the foreseeable future, including those with dementia or psychiatric disorders—14.4% of all cases in 2020–2021 [19]. The figures are calculated for each line of euthanasia counts by year, age, gender, and language, and are then used as offset in the model. Demographic data do not include information on language. To tackle this issue, the French-speaking population was calculated as the sum of the population residing in Wallonia and 90% of the population in Brussels and the Dutch-speaking as the sum of the Flanders residents and 10% of the Brussels population, reflecting the Belgian language repartition. This 10% figure is an estimate of the Dutch-speaking population residing in Brussels. In 2001, 8.4% of Brussels residents were born in Flanders, and this proportion has slightly increased over time [31].

### Analyses

The dataset contains 126,720 lines in total, with each line reporting the number of euthanasia cases (including zero counts). These lines represent combinations of population characteristics (gender [2] × language [2] × age group [8] = 32) and euthanasia characteristics (reason [3] × place of death [5] × type of suffering [3] × term of death [2] × basis of euthanasia [2] = 180) across 22 years.

To examine trends in euthanasia case counts across demographic and clinical subgroups, we employed zero-inflated negative binomial (ZINB) regression models [31] using the glmmTMB package in R [32]. Original analyses were made using Poisson Regression modelling. We used ZINB following reviewers’ recommendations. The initial Poisson regression revealed substantial overdispersion—where the variance significantly exceeded the mean—violating the core assumptions of the Poisson model. Specifically, the dispersion ratio was considerably greater than 1 (see S1 File). In contrast, negative binomial models, based on the “nbinom2” parameterisation, substantially reduced overdispersion, indicating improved model fit. This formulation models the variance as a quadratic function of the mean: Var(*Y*) = *μ* + *μ*^2^/*θ*, making it well-suited to count data with moderate to high overdispersion.

Because excess zeros in the data due to low cases among the population, we specified a zero-inflated component in the models [33,34]. Specifically, we included age group, gender, and language group as predictors in the zero-inflation formula to account for differential probabilities of observing structural zeros across subpopulations. This approach allowed us to model both the count process and the zero-generating process simultaneously, offering a more nuanced and accurate representation of the data-generating mechanisms. We only report results from the main model in this study.

Our primary count model included a two-way interaction between year (treated as a continuous variable) and reason for euthanasia, controlling for age-groups, gender, and languages. To account for variability in the underlying population at risk across different strata, we estimated two versions of each model: one without an offset term (model 1)—interpretable as modelling expected case counts—and one including a log-offset for the relevant population denominator (model 2), thereby modelling incidence rates.

In Model 1 (without offset), the exponentiated coefficients represent multiplicative changes in the expected number of euthanasia cases (count) associated with a one-unit increase in the predictor variables. For example, if the exponentiated coefficient for year is 1.05, this suggests that each additional year is associated with a 5% increase in the expected euthanasia count, assuming all other variables remain constant. Similarly, categorical predictors (e.g., gender or age groups) compare the expected counts between groups. Importantly, this model treats the outcome purely as a count, without accounting for differences in population size.

In Model 2 (with offset), the exponentiated coefficients instead reflect changes in the rate of euthanasia per unit of the offset variable (i.e., sub-groups population size). Here, a coefficient of 1.05 for year would mean that each additional year corresponds to a 5% increase in the euthanasia rate (number of cases relative to the offset), holding other variables constant. This model is more appropriate when the underlying population size influences the observed counts and to isolate the effects of predictors on the rate rather than the raw count.

Similarity in estimates between the two models would suggest that the sub-groups populations have limited variability across observations, meaning population size does not substantially modify the observed relationships. If the offset variable is highly variable, the coefficients in Model 2 would adjust for these disparities, potentially diverging from Model 1.

To explore how temporal trends in euthanasia case counts might differ across demographic or clinical subgroups, we further estimated extended models incorporating three-way interactions between year, reason for euthanasia, and each covariate of interest controlling only for socio-demographic variables (i.e., gender, region, age-group). For transparency, we calculated 95% confidence intervals (CIs) for each model. Additionally, due to low counts in some sub-categories, the 95% CIs could not be calculated because of convergence issues.

This study is reported as per the Strengthening the Reporting of Observational Studies in Epidemiology (STROBE) guideline (S1 Checklist).

## Results

Table 1 exhibits the number of euthanasia cases by reason, distinguishing between dementia, psychiatric disorders, and other causes from 2002 to 2023. To ensure anonymity and avoid low counts (<3), the years 2002–2007 (the first 6 years following legalization) are grouped into a single category. Euthanasia cases for dementia or psychiatric disorders are reported 100 times less frequently than for other reasons, such as cancer or comorbidities, while following a similar trend to these more common cases. Over the selected period, psychiatric disorders and dementia represent 1.27% and 0.92% of all cases, respectively, and followed similar trends to the overall number of cases.

**Table 1:** Yearly count of reported cases of euthanasia by reason.

	Terminal illness		Dementia		Psychiatric disorders		Total
Year	Count	*Percent*	Count	*Percent*	Count	*Percent*	
2002–2007	1,908	*99.38*	6	*0.31*	6	*0.31*	1,920
2008	695	*98.86*	5	*0.71*	3	*0.43*	703
2009	804	*98.17*	6	*0.73*	9	*1.10*	819
2010	928	*98.10*	8	*0.85*	10	*1.06*	946
2011	1,099	*97.52*	14	*1.24*	14	*1.24*	1,127
2012	1,384	*96.92*	14	*0.98*	30	*2.10*	1,428
2013	1,760	*97.24*	13	*0.72*	37	*2.04*	1,810
2014	1,866	*96.78*	18	*0.93*	44	*2.28*	1,928
2015	1,959	*96.88*	20	*0.99*	43	*2.13*	2,022
2016	1,988	*98.03*	11	*0.54*	29	*1.43*	2,028
2017	2,273	*98.27*	14	*0.61*	26	*1.12*	2,313
2018	2,303	*97.63*	22	*0.93*	34	*1.44*	2,359
2019	2,608	*98.16*	26	*0.98*	23	*0.87*	2,657
2020	2,401	*98.20*	23	*0.94*	21	*0.86*	2,445
2021	2,647	*98.11*	27	*1.00*	24	*0.89*	2,698
2022	2,898	*97.71*	42	*1.42*	26	*0.88*	2,966
2023	3,334	*97.40*	41	*1.20*	48	*1.40*	3,423
Total	32,855	*97.81*	310	*0.92*	427	*1.27*	33,592

### (R.Q.1) How do trends in euthanasia cases involving psychiatric conditions and cognitive disorders compare with those for other reasons, such as terminal illness, and how does this comparison change when demographic composition is taken into account?

We present the exponentialized coefficients, i.e., the counts (without demographic offset) and rates (with demographic offset) and 95%CI in Table 2. The main model includes a two-way multiplicative interaction term between the year cases were reported and reason for euthanasia. Other models use a three-way interaction including, respectively, gender, region, basis for euthanasia (i.e., whether the request was made in advance or not), expected term of death, type of suffering and place of death. The full results are shown in S2–S8 Files.

**Table 2:** Relative risks (RR) and Incidence Ratios (IR) of the two-way and three-way interactions.

	Count		Rate			Count		Rate	
Main model	Exp(B)	*(95% CI)*	Exp(B)	*95%CI*	Type of suffering (Ref.: physical)	Exp(B)	*(95% CI)*	Exp(B)	*95%CI*
Year	1.075	*(1.06; 1.091)*	1.044	*(1.036; 1.053)*	Year	1.043	*(1.032; 1.054)*	1.025	*(1.014; 1.036)*
Dementia	0.028	*(0.018; 0.044)*	0.024	*(0.015; 0.037)*	Dementia	0.021	*(0.005; 0.082)*	0.021	*(0.005; 0.081)*
Psychiatric disorders	0.071	*(0.05; 0.102)*	0.065	*(0.045; 0.095)*	Psychiatric disorders	0.016	*(0.003; 0.08)*	0.016	*(0.003; 0.079)*
Year × Dementia	1.032	*(1.003; 1.062)*	1.044	*(1.014; 1.074)*	Mental	0.247	*(0.189; 0.323)*	0.244	*(0.187; 0.319)*
Year × Psychiatric disorders	1.004	*(0.98; 1.029)*	1.016	*(0.99; 1.042)*	Both	2.304	*(1.939; 2.738)*	2.302	*(1.936; 2.738)*
**Gender** (ref.: Female)					Year × Dementia	0.937	*(0.846; 1.037)*	0.936	*(0.846; 1.036)*
Year	1.077	*(1.066; 1.089)*	1.071	*(1.058; 1.084)*	Year × Psychiatric disorders	0.929	*(0.823; 1.047)*	0.928	*(0.824; 1.046)*
Dementia	0.03	*(0.016; 0.055)*	0.035	*(0.019; 0.062)*	Year × mental	0.897	*(0.879; 0.916)*	0.898	*(0.88; 0.916)*
Psychiatric disorders	0.092	*(0.058; 0.148)*	0.101	*(0.064; 0.157)*	Year × both	1.026	*(1.014; 1.038)*	1.026	*(1.014; 1.038)*
Male	1.459	*(1.175; 1.812)*	1.659	*(1.382; 1.992)*	Dementia × mental	7.706	*(1.69; 35)*	7.932	*(1.75; 35)*
Year × Dementia	1.026	*(0.987; 1.068)*	1.017	*(0.979; 1.057)*	Psychiatric disorders × mental	39.208	*(7.318; 210)*	40.198	*(7.567; 213)*
Year × Psychiatric disorders	1.016	*(0.984; 1.049)*	1.007	*(0.976; 1.038)*	Dementia × both	0.659	*(0.139; 3.126)*	0.665	*(0.141; 3.131)*
Year × Male	0.975	*(0.962; 0.988)*	0.988	*(0.976; 1)*	Psychiatric disorders × both	1.379	*(0.242; 7.857)*	1.392	*(0.246; 7.864)*
Dementia × Male	0.588	*(0.237; 1.461)*	0.679	*(0.283; 1.63)*	Year × Dementia × mental	1.283	*(1.149; 1.432)*	1.281	*(1.148; 1.43)*
Psychiatric disorders × Male	0.387	*(0.173; 0.867)*	0.41	*(0.19; 0.884)*	Year × Psychiatric disorders × mental	1.252	*(1.104; 1.42)*	1.252	*(1.105; 1.419)*
Year × Dementia × Male	1.038	*(0.98; 1.101)*	1.025	*(0.97; 1.084)*	Year × Dementia × both	1.092	*(0.976; 1.222)*	1.091	*(0.976; 1.22)*
Year × Psychiatric disorders × Male	1.005	*(0.952; 1.06)*	0.997	*(0.947; 1.05)*	Year × Psychiatric disorders × both	1.071	*(0.942; 1.219)*	1.071	*(0.943; 1.217)*
**Region** (ref.: FR)					**Place** (ref.: home)				
Year	1.098	*(1.082; 1.115)*	1.092	*(1.078; 1.106)*	Year	1.152	*N/C*	1.07	*(1.058; 1.082)*
Dementia	0.014	*(0.005; 0.042)*	0.014	*(0.005; 0.042)*	Dementia	0.037	*N/C*	0.026	*(0.013; 0.051)*
Psychiatric disorders	0.002	*(0; 0.011)*	0.002	*(0; 0.011)*	Psychiatric disorders	0.093	*N/C*	0.085	*(0.049; 0.15)*
NL	5.196	*(4.246; 6.358)*	3.403	*(2.804; 4.129)*	Hospital	1.452	*N/C*	1.474	*(1.217; 1.787)*
year × Dementia	1.068	*(1.002; 1.139)*	1.068	*(1.002; 1.138)*	Palliative care	0.005	*N/C*	0.003	*(0.002; 0.005)*
year × Psychiatric disorders	1.175	*(1.059; 1.303)*	1.173	*(1.058; 1.3)*	Nursing home	0.121	*N/C*	0.098	*(0.078; 0.125)*
year × NL	0.964	*(0.952; 0.976)*	0.96	*(0.949; 0.972)*	Other	0.053	*N/C*	0.042	*(0.031; 0.059)*
Dementia × NL	2.099	*(0.641; 6.875)*	2.165	*(0.662; 7.081)*	Year × Dementia	1.018	*N/C*	1.041	*(0.996; 1.088)*
Psychiatric disorders × NL	51.977	*(7.634; 353.8)*	50.855	*(7.513; 344.2)*	Year × Psychiatric disorders	1.005	*N/C*	1.021	*(0.982; 1.061)*
Year × Dementia × NL	0.967	*(0.9; 1.038)*	0.963	*(0.897; 1.034)*	Year × Hospital	0.964	*N/C*	0.962	*(0.948; 0.975)*
Year × Psychiatric disorders × NL	0.863	*(0.775; 0.96)*	0.865	*(0.777; 0.962)*	Year × Palliative care	1.144	*N/C*	1.184	*(1.152; 1.218)*
**Basis** (Ref.: not advanced)					Year × Nursing home	1.06	*N/C*	1.061	*(1.044; 1.078)*
Year	1.134	*N/C*	1.042	*(1.034; 1.051)*	Year × Other	0.98	*N/C*	0.994	*(0.973; 1.017)*
Dementia	0.031	*N/C*	0.022	*(0.014; 0.035)*	Dementia × Hospital	0.572	*N/C*	0.591	*(0.206; 1.692)*
Psychiatric disorders	0.077	*N/C*	0.069	*(0.047; 0.1)*	Psychiatric disorders × Hospital	0.458	*N/C*	0.445	*(0.182; 1.087)*
Advanced	0.047	*N/C*	0.041	*(0.032; 0.054)*	Dementia × Palliative care	0	*N/C*	0	*N/C*
Year × Dementia	1.022	*N/C*	1.049	*(1.018; 1.08)*	Psychiatric disorders × Palliative care	0	*N/C*	0	*(0; 72038.79)*
Year × Psychiatric disorders	0.996	*N/C*	1.013	*(0.988; 1.04)*	Dementia × Nursing home	2.793	*N/C*	3.251	*(0.96; 11.003)*
Year × advanced	0.912	*N/C*	0.923	*(0.905; 0.941)*	Psychiatric disorders × Nursing home	2.446	*N/C*	2.401	*(0.834; 6.908)*
Dementia × advanced	4.104	*N/C*	5.508	*(1.191; 25.4)*	Dementia × Other	0.703	*N/C*	0.796	*(0.06; 10.59)*
Psychiatric disorders × advanced	0	*N/C*	0.334	*N/C*	Psychiatric disorders × Other	1.057	*N/C*	0.921	*(0.214; 3.967)*
Year × Dementia × advanced	0.978	*N/C*	0.956	*(0.855; 1.07)*	Year × Dementia × Hospital	1.004	*N/C*	1.005	*(0.938; 1.077)*
Year × Psychiatric disorders × advanced	0.616	*N/C*	0.002	*N/C*	Year × Psychiatric disorders × Hospital	0.993	*N/C*	0.99	*(0.93; 1.054)*
**Term of death** Ref.: short-term)					Year × Dementia × Palliative care	18,325	*N/C*	20,316	*N/C*
Year	1.137	*(1.128; 1.145)*	1.068	*(1.042; 1.094)*	Year × Psychiatric disorders × Palliative care	3.37	*N/C*	3.343	*(0.556; 20.09)*
Dementia	0.018	*(0.009; 0.039)*	0.016	*(0.007; 0.035)*	Year × Dementia × Nursing home	0.958	*N/C*	0.96	*(0.889; 1.036)*
Psychiatric disorders	0.007	*(0.002; 0.023)*	0.006	*(0.002; 0.021)*	Year × Psychiatric disorders × Nursing home	0.913	*N/C*	0.915	*(0.852; 0.983)*
Not short-term	0.073	*(0.062; 0.086)*	0.068	*(0.056; 0.083)*	Year × Dementia × Other	1.066	*N/C*	1.059	*(0.908; 1.236)*
Year × Dementia	0.939	*(0.89; 0.992)*	0.951	*(0.9; 1.005)*	Year × Psychiatric disorders × Other	1.089	*N/C*	1.092	*(0.997; 1.196)*
Year × Psychiatric disorders	0.942	*(0.868; 1.023)*	0.954	*(0.878; 1.037)*					
Year × Not short-term	1.054	*(1.042: 1.067)*	1.058	*(1.044; 1.072)*					
Dementia × Not short-term	16.143	*(6.4; 40)*	16.971	*(6.6; 43.1)*					
Psychiatric disorders × Not short-term	125.2	*(36.3; 430.9)*	146.462	*(41.5; 515)*					
Year × Dementia × Not short-term	1.056	*(0.991; 1.125)*	1.053	*(0.988; 1.123)*					
Year × Psychiatric disorders × Not short-term	1.023	*(0.939; 1.116)*	1.016	*(0.931; 1.109)*					

Note: N/C denotes non-convergence.

The main model investigated the two-way interaction between the reason for euthanasia and time, comparing trends for psychiatric disorders and dementia to “other” reasons, which serve as the reference category. The findings indicate that euthanasia for dementia has shown a modest but significant annual increase relative to “other” reasons. The interaction term between “year” and “dementia” cases is 1.03 in the model without offset (year × dementia, count = 1.03 [95%CI: 1.00; 1.07]) and 1.04 in the model with offset (rate = 1.04 [95%CI: 1.01; 1.07]). This result suggests that euthanasia for dementia-related cases is becoming relatively more common over time compared to “other” reasons. In contrast, the trend for psychiatric disorders remains relatively stable, although a slight increase is observed when the model includes an offset (year × psychiatric disorders, RR = 1.00 [95%CI: 0.98; 1.03]; IR = 1.02 [95%CI: 0.99; 1.04]). This finding highlights that psychiatric disorders have not followed the same increasing trajectory as dementia when compared to “other” reasons.

**Fig 1:**
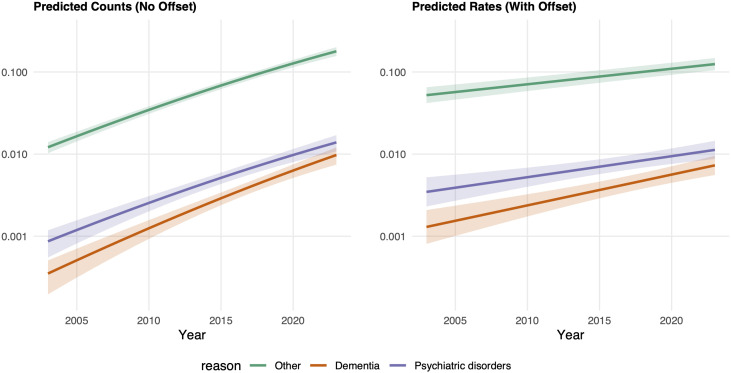
Predicted counts and rates of euthanasia for dementia, psychiatric disorders and other causes by year (2003–2023), negative binomial regression (and 95%CI).

Fig 1 plots the expected counts and rates of the main model. In the left panel, the predicted counts (without offset) show a curved pattern over time because they reflect both changes in the underlying risk of the event and fluctuations in the population size. In contrast, the right panel shows predicted rates (with offset), which adjust for population size and therefore isolate the trend in the individual risk of the event. The difference between predicted rates with and without adjustment for population composition and change suggests that population change may partially underlie changes observed in euthanasia prevalence. Furthermore, the rate of change is relatively similar when comparing euthanasia for psychiatric disorders to the other types of causes, but the increase is sharper for dementia.

We have replicated the main model using year as a factor variable (i.e., calculating the rates separately for each time points) with no significant difference observed as can be seen in S9 File).

### (R.Q.2) Are distinct patterns observable across population sub-groups?

The three-way model looking at gender revealed notable differences in trends. We observe overall lower prevalence in male in euthanasia for dementia and psychiatric disorders (Dementia × Male, rate: 0.68 [95%CI: 0.28; 1.63]—Psychiatric disorders × Male: 0.41 [95%CI: 0.19; 0.88]). The trend was relatively stable for psychiatric disorders, but the prevalence of euthanasia for dementia among male patients has increased over time (Year * Dementia × Male: 1.02 [95%CI: 0.97; 1.08]).

Regional patterns between the Dutch-speaking region (NL—the Flanders) and the French-speaking region (Wallonia–Brussels) also emerged. Dementia-related euthanasia are more often observed in the Flanders (dementia × NL, rate = 2.16 (95%CI: 0.66; 7.08)), but the rate for psychiatric disorders is particularly high (Psychiatric disorders × NL, rate: 50.85 (95%CI: 7.51; 344.2) indicating that cases of euthanasia for psychiatric disorders and dementia are less often observed in Wallonia and Brussels. However, the rate of change is lower for the Flanders compared to Wallonia–Brussels over the selected period (Year × Dementia × NL, rate: 0.96 [95%CI: 0.90; 1.03] − Year × Psychiatric disorders × NL, rate: 0.865 [95%CI: 0.78; 0.96].

Analyses of the basis of euthanasia (i.e., advanced or not) cause non-convergence issues because of the low count observed for dementia and psychiatric disorder cases. Estimates show a higher rate for dementia with advanced request when looking at the two-way interaction, but this is balanced by below-1 rates for the main term and the three-way interaction. In other words, neither dementia nor psychiatric disorders are associated with advanced request and this has not changed over the selected period.

Mental suffering was higher in cases of euthanasia for dementia and psychiatric disorders compared to non-psychiatric/dementia cases (dementia × mental suffering, rate = 7.93 [95%CI: 1.75; 35]) − Psychiatric disorders × mental suffering, rate = 40.198 [95%CI: 7.57; 213]).

The three-way interaction with the place of death is less easy to interpret given the low count observed of euthanasia for psychiatric disorders and dementia. What can be observed is that euthanasia for both psychiatric and dementia reasons is more prevalent at home than in hospital or palliative care settings. Data indicate a potential increase of euthanasia for dementia and psychiatric disorders in palliative care, but low counts lead to convergence issue and this trend should be taken with caution. Unsurprisingly, euthanasia for dementia is more likely to occur in a nursing home than at home (rate = 3.25 [95%CI: 0.96; 11.00]), but this is also the case of euthanasia for psychiatric disorders (rate = 2.40 [95%CI: 0.83; 6.91]).

**Fig 2:**
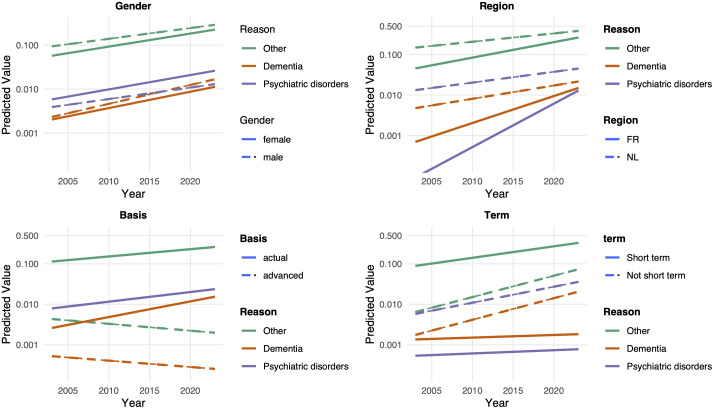
Predicted Incidence Ratios of euthanasia by reason, year and gender, region, basis and term, 2003–2023.

To visualise these trends, Fig 2 presents the expected rates of euthanasia by gender, region, basis of request, and expected term of death.

A clear divergence emerges in terms of gender. Euthanasia due to psychiatric disorders has been consistently more prevalent among women since the beginning of the observed period, and the gap with men has slightly widened over time. In contrast, cases involving dementia were initially slightly more common among men, and this disparity has increased over time.

These cases have been more prevalent in the Dutch-speaking community than the French-speaking community, although this regional difference has diminished over time. Furthermore, we observed that most of the increase in psychiatric and dementia-related euthanasia occurred in the French-speaking community.

Euthanasia for psychiatric disorders and dementia based on advance requests has not increased significantly. The rise in cases is largely attributable to actual (non-advance) requests.

## Discussion

Recent debates on the implementation of assisted dying in the United Kingdom and France have highlighted the distinction between euthanasia for terminal- and non-terminal causes, with both these countries considering implementation of euthanasia for terminal illnesses only, excluding patients with psychiatric disorders or degenerative conditions [35,36]. In contrast, Belgium euthanasia law has allowed euthanasia for non-terminal and non-physical illnesses from its inception in 2002, allowing us to compare rates in euthanasia for terminal illnesses, psychiatric disorders, and dementia.

These regulations would *de facto* exclude patients with psychiatric disorders or degenerative conditions such as dementia who may wish to request ADE. Opponents of such rights often argue that a narrowly defined assisted dying law would inevitably expand to include non-terminal illnesses, resulting in an over increase in cases. The Belgian experience, however, offers contextual insights into these concerns. From the outset, the Belgian law has allowed euthanasia for non-terminal and non-physical illnesses under specific safeguards, enforced by medical practitioners and monitored by the FCCEE.

Our study shows a significant, but still modest, increase in euthanasia for dementia. Furthermore, differences across genders and linguistic regions have declined over time. Trends show gradual implementation, with a steady yearly rate of change for psychiatric conditions and a slightly sharper but still modest increase for dementia. During the first 5 years following the implementation of the regulation, cases of euthanasia for psychiatric disorders and dementia remained infrequent. Differences in access across genders and linguistic regions have narrowed over time. In 2023, psychiatric disorders and dementia accounted for 1.4% and 1.2% of all cases, respectively.

Requests for euthanasia made in advance are rare and mainly associated with non-psychiatric and non-dementia conditions. Euthanasia on patients not expected to die in the foreseeable future have risen more sharply among those with ‘other” conditions groups rather than among those with psychiatric conditions. However, we observe a sharp increase in euthanasia for patients with dementia not expected to die in the foreseeable future. As cases remain low, it is difficult to draw any conclusion on future trends, but euthanasia seems to become more common among patients with dementia, living in care facilities, and not expected to die within a year.

This study has two major limitations.

A first limitation is about the data. Studies focussing on the Belgian context rely on administrative data collected by the FCCEE. The absence of social security identifiers in euthanasia records prevents data linkage with socio-economic information, while a lack of geographic data precludes sub-regional comparisons. The FCCEE acts as a commission controlling individual cases, not as an organism controlling euthanasia as a public policy. Critics of ADE regulations often focus on both the safeguards that should protect individuals and the trends that affect populations and sub-populations. This well-known distinction in public health between populations and individuals [37] reflects in the way data are collected in Belgium and policymakers should consider proactive data planning, including establishing data collection protocols, securing patient consent, ensuring data anonymization, enabling data linkage, and committing to independent research. Furthermore, this study focuses on administrative trends and does not address clinical, psychological, or qualitative dimensions.

A second limitation concerns the methods used to analyse ADE figures. Compared to our previous work where we used a Poisson regression modelling [4], this study uses a zero-inflated binomial model. Count data exhibit excess zeros that are not well accounted for by the Poisson distribution and the binomial model is appropriate when counts are bounded by a known total. We observe no significant differences in trends across both models but the Poisson distribution tends to reduce the trends observed of psychiatric disorders while a relative increase is observed in this study. Analyses on small counts may be sensitive to the model used, and we encourage researchers to compare different models to strengthen their findings.

Concerns have been raised, both in parliamentary debates [38] and within professional medical bodies, that permitting euthanasia in limited cases (e.g., terminal illness) may gradually lead to less justified applications, and whether safeguards are effectively maintained when euthanasia is extended to non-terminal conditions or psychiatric disorders [39]. While we acknowledge that empirical data cannot resolve ethical and normative questions, our findings demonstrate that euthanasia for psychiatric and cognitive conditions, which has been legally permissible in Belgium since 2002, has not substantially increased between 2002 and 2023. These findings can add to the discussion of end-of-life issues and their inherent complexities.

## Supporting information

S1 File Comparison between Poisson and negative binomial models for the main outcomes. (DOCX)

S2 File Zero-inflated negative binomial regression of Reason by Year (two-way interaction) and Predicted counts and rates. (DOCX)

S3 File Zero-inflated negative binomial regression of Reason by Year and Gender (three-way interaction) and Predicted counts and rates. (DOCX)

S4 File Zero-inflated negative binomial regression of Reason by Year and Region (three-way interaction) and Predicted relative risks and incidence ratios. (DOCX)

S5 File Zero-inflated negative binomial regression of Reason by Year and basis (three-way interaction) and Predicted counts and rates. (DOCX)

S6 File Zero-inflated negative binomial regression of Reason by Year and expected term of death (three-way interaction) and Predicted counts and rates. (DOCX)

S7 File Zero-inflated negative binomial regression of Reason by Year and type of suffering (three-way interaction) and Predicted relative risks and incidence ratios. (DOCX)

S8 File Zero-inflated negative binomial regression of Reason by Year and place of death (three-way interaction). (DOCX)

S9 File Zero-inflated negative binomial regression, predicted counts and rates of Reason by Year (factor) (two-way interaction). (DOCX)

S1 Checklist DOI: 10.1016/j.jclinepi.2007.11.008. (DOCX)

## Abbreviations

ADE: Assisted dying and euthanasia
CIs: confidence intervals
FCCEE: Federal Control and Evaluation Commission on Euthanasia
STROBE: Strengthening the Reporting of Observational Studies in Epidemiology
ZINB: zero-inflated negative binomial
