# Supplementary material for: Trends in assisted dying among patients with psychiatric disorders and dementia in Belgium: A health registry study
Source: PLoS Med. 2025 Nov 19;22(11):e1004522. doi: 10.1371/journal.pmed.1004522 (PMC12646481; doi:10.1371/journal.pmed.1004522)
Supplement: S1 File — (DOCX) [file pmed.1004522.s001.docx]

# S.1. Comparison between Poisson and negative binomial models for the main outcomes

| Model | **Poisson** |  | **Negative binomial (NB)** |
| --- | --- | --- | --- |
| R Package | glmmTMB |  | glmmTMB |
| Formula | glmmTMB( count ~ year*reason + Age group + Gender + Language + basis + suffering + term + Place + offset(log(populations)),  + family = poisson + data = data |  | glmmTMB(count ~ year*reason + Age group + Gender + Language + basis + suffering + term + Place + offset(log(populations)),  + family = nbinom2 + data = data Note: NB2 (variance = μ + μ²/θ) |
| Overdispersion ratio | 5.739 (p-value < 0.001) |  | 1.360 (p-value < 0.001) |
| Df | 23 |  | 24 |
| AIC | 60222.95 |  | 39755.36 |
| BIC | 60386.57 |  | 39926.09 |
| Likelihood ratio test | \| LogLik \| Chisq \| Pr (>Chisq) \| \| --- \| --- \| --- \| \| -30088 \|  \|  \| |  | \| LogLik \| Chisq \| Pr (>Chisq) \| \| --- \| --- \| --- \| \| -19854 \| 20470 \| 0.000 (***) \| |
|  | 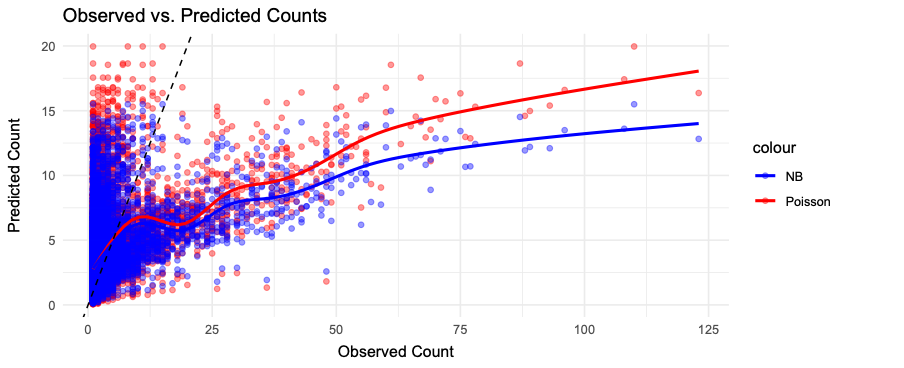 | | |
