# Supplementary material for: Trends in assisted dying among patients with psychiatric disorders and dementia in Belgium: A health registry study
Source: PLoS Med. 2025 Nov 19;22(11):e1004522. doi: 10.1371/journal.pmed.1004522 (PMC12646481; doi:10.1371/journal.pmed.1004522)
Supplement: S2 File — (DOCX) [file pmed.1004522.s002.docx]

# S.2. Zero-inflated negative binomial regression of Reason by Year (two-way interaction)

| Variable | No offset | 95%CI + | 95%CI - | With offset | 95%CI + | 95%CI - |
| --- | --- | --- | --- | --- | --- | --- |
| (Intercept) | 0.061 | 0.050 | 0.075 | 0.000 | 0.000 | 0.000 |
| Age group= 15-29 | 0.024 | 0.014 | 0.043 | 0.027 | 0.020 | 0.036 |
| Age group= 30-39 | 0.189 | 0.125 | 0.284 | 0.116 | 0.094 | 0.143 |
| Age group= 40-49 | 0.411 | 0.328 | 0.515 | 0.338 | 0.286 | 0.401 |
| Age group= 60-69 | 1.732 | 1.481 | 2.025 | 2.374 | 2.079 | 2.710 |
| Age group= 70-79 | 1.754 | 1.490 | 2.065 | 3.906 | 3.391 | 4.500 |
| Age group= 80-89 | 1.528 | 1.274 | 1.832 | 6.645 | 5.708 | 7.736 |
| Age group= 90+ | 0.568 | 0.453 | 0.712 | 12.704 | 10.765 | 14.993 |
| Gender= male | 0.943 | 0.870 | 1.023 | 1.230 | 1.144 | 1.324 |
| Language= NL | 3.162 | 2.885 | 3.466 | 1.927 | 1.778 | 2.088 |
| Reason= Dementia | 0.028 | 0.018 | 0.044 | 0.024 | 0.015 | 0.037 |
| Reason= Psychiatric disorders | 0.071 | 0.050 | 0.102 | 0.065 | 0.045 | 0.095 |
| year | 1.075 | 1.060 | 1.091 | 1.044 | 1.036 | 1.053 |
| Year * reason= Dementia | 1.032 | 1.003 | 1.062 | 1.044 | 1.014 | 1.074 |
| Year* reason= Psychiatric disorders | 1.004 | 0.980 | 1.029 | 1.016 | 0.990 | 1.042 |

## Predicted counts and rates
