# Supplementary material for: Trends in assisted dying among patients with psychiatric disorders and dementia in Belgium: A health registry study
Source: PLoS Med. 2025 Nov 19;22(11):e1004522. doi: 10.1371/journal.pmed.1004522 (PMC12646481; doi:10.1371/journal.pmed.1004522)
Supplement: S3 File — (DOCX) [file pmed.1004522.s003.docx]

# S.3. Zero-inflated negative binomial regression of Reason by Year and Gender (three-way interaction)

| Variable | No offset | 95%CI + | 95%CI - | With offset | 95%CI + | 95%CI - |
| --- | --- | --- | --- | --- | --- | --- |
| (Intercept) | 0.032 | 0.026 | 0.039 | 0.000 | 0.000 | 0.000 |
| Age group= 15-29 | 0.034 | 0.026 | 0.043 | 0.024 | 0.012 | 0.048 |
| Age group= 30-39 | 0.108 | 0.091 | 0.128 | 0.206 | 0.145 | 0.290 |
| Age group= 40-49 | 0.320 | 0.278 | 0.368 | 0.417 | 0.338 | 0.514 |
| Age group= 60-69 | 1.988 | 1.763 | 2.242 | 2.058 | 1.770 | 2.392 |
| Age group= 70-79 | 2.379 | 2.116 | 2.675 | 2.966 | 2.555 | 3.443 |
| Age group= 80-89 | 2.312 | 2.054 | 2.603 | 4.637 | 3.971 | 5.414 |
| Age group= 90+ | 0.914 | 0.801 | 1.043 | 8.202 | 6.849 | 9.822 |
| Gender= male | 1.459 | 1.175 | 1.812 | 1.659 | 1.382 | 1.992 |
| Language= NL | 3.175 | 2.956 | 3.409 | 1.973 | 1.809 | 2.152 |
| Reason= Dementia | 0.030 | 0.016 | 0.055 | 0.035 | 0.019 | 0.062 |
| Reason= Dementia * Gender= male | 0.588 | 0.237 | 1.461 | 0.679 | 0.283 | 1.630 |
| Reason= Psychiatric disorders | 0.092 | 0.058 | 0.148 | 0.101 | 0.064 | 0.157 |
| Reason= Psychiatric disorders * Gender= male | 0.387 | 0.173 | 0.867 | 0.410 | 0.190 | 0.884 |
| year | 1.077 | 1.066 | 1.089 | 1.071 | 1.058 | 1.084 |
| Year * Gender= male | 0.975 | 0.962 | 0.988 | 0.988 | 0.976 | 1.000 |
| Year * reason= Dementia | 1.026 | 0.987 | 1.068 | 1.017 | 0.979 | 1.057 |
| Year * reason= Dementia * Gender= male | 1.038 | 0.980 | 1.101 | 1.025 | 0.970 | 1.084 |
| Year * reason= Psychiatric disorders | 1.016 | 0.984 | 1.049 | 1.007 | 0.976 | 1.038 |
| Year * reason= Psychiatric disorders * Gender= male | 1.005 | 0.952 | 1.060 | 0.997 | 0.947 | 1.050 |

## Predicted counts and rates by gender
