# Supplementary material for: Trends in assisted dying among patients with psychiatric disorders and dementia in Belgium: A health registry study
Source: PLoS Med. 2025 Nov 19;22(11):e1004522. doi: 10.1371/journal.pmed.1004522 (PMC12646481; doi:10.1371/journal.pmed.1004522)
Supplement: S4 File — (DOCX) [file pmed.1004522.s004.docx]

# S.4. Zero-inflated negative binomial regression of Reason by Year and Region (three-way interaction)

| Variable | No offset | 95%CI + | 95%CI - | With offset | 95%CI + | 95%CI - |
| --- | --- | --- | --- | --- | --- | --- |
| (Intercept) | 0.044 | 0.035 | 0.055 | 0 | 0 | 0 |
| Age group= 15-29 | 0.027 | 0.015 | 0.047 | 0.026 | 0.013 | 0.053 |
| Age group= 30-39 | 0.176 | 0.118 | 0.262 | 0.2 | 0.143 | 0.279 |
| Age group= 40-49 | 0.396 | 0.318 | 0.495 | 0.409 | 0.333 | 0.502 |
| Age group= 60-69 | 1.79 | 1.532 | 2.091 | 2.124 | 1.828 | 2.467 |
| Age group= 70-79 | 1.822 | 1.551 | 2.139 | 3.064 | 2.645 | 3.549 |
| Age group= 80-89 | 1.596 | 1.336 | 1.906 | 4.759 | 4.092 | 5.536 |
| Age group= 90+ | 0.595 | 0.479 | 0.739 | 8.364 | 7.031 | 9.95 |
| Gender= male | 0.952 | 0.878 | 1.033 | 1.339 | 1.238 | 1.448 |
| Language= NL | 5.196 | 4.246 | 6.358 | 3.403 | 2.804 | 4.129 |
| Reason= Dementia | 0.014 | 0.005 | 0.042 | 0.014 | 0.005 | 0.042 |
| Reason= Dementia * Language= NL | 2.099 | 0.641 | 6.875 | 2.165 | 0.662 | 7.081 |
| Reason= Psychiatric disorders | 0.002 | 0 | 0.011 | 0.002 | 0 | 0.011 |
| Reason= Psychiatric disorders * Language= NL | 51.977 | 7.634 | 353.881 | 50.855 | 7.513 | 344.211 |
| year | 1.098 | 1.082 | 1.115 | 1.092 | 1.078 | 1.106 |
| Year * Language= NL | 0.964 | 0.952 | 0.976 | 0.96 | 0.949 | 0.972 |
| Year * reason= Dementia | 1.068 | 1.002 | 1.139 | 1.068 | 1.002 | 1.138 |
| Year * reason= Dementia * Language= NL | 0.967 | 0.9 | 1.038 | 0.963 | 0.897 | 1.034 |
| Year * reason= Psychiatric disorders | 1.175 | 1.059 | 1.303 | 1.173 | 1.058 | 1.3 |
| Year * reason= Psychiatric disorders * Language= NL | 0.863 | 0.775 | 0.96 | 0.865 | 0.777 | 0.962 |

## Predicted relative risks and incidence ratios by region
