# Supplementary material for: Trends in assisted dying among patients with psychiatric disorders and dementia in Belgium: A health registry study
Source: PLoS Med. 2025 Nov 19;22(11):e1004522. doi: 10.1371/journal.pmed.1004522 (PMC12646481; doi:10.1371/journal.pmed.1004522)
Supplement: S5 File — (DOCX) [file pmed.1004522.s005.docx]

# S.5. Zero-inflated negative binomial regression of Reason by Year and basis (three-way interaction)

| Variable | No offset | 95%CI + | 95%CI - | With offset | 95%CI + | 95%CI - |
| --- | --- | --- | --- | --- | --- | --- |
| (Intercept) | 0.054 | N/C | N/C | 0 | 0 | 0 |
| Age group= 15-29 | 0.051 | N/C | N/C | 0.026 | 0.02 | 0.036 |
| Age group= 30-39 | 0.214 | N/C | N/C | 0.114 | 0.093 | 0.14 |
| Age group= 40-49 | 0.443 | N/C | N/C | 0.335 | 0.284 | 0.394 |
| Age group= 60-69 | 1.533 | N/C | N/C | 2.369 | 2.084 | 2.693 |
| Age group= 70-79 | 1.402 | N/C | N/C | 3.771 | 3.31 | 4.296 |
| Age group= 80-89 | 1.121 | N/C | N/C | 6.356 | 5.549 | 7.281 |
| Age group= 90+ | 0.354 | N/C | N/C | 12.185 | 10.501 | 14.139 |
| Basis= advanced | 0.047 | N/C | N/C | 0.041 | 0.032 | 0.054 |
| Gender= male | 0.843 | N/C | N/C | 1.229 | 1.148 | 1.317 |
| Language= NL | 3.46 | N/C | N/C | 1.805 | 1.672 | 1.949 |
| Reason= Dementia | 0.031 | N/C | N/C | 0.022 | 0.014 | 0.035 |
| Reason= Dementia * basis= advanced | 4.104 | N/C | N/C | 5.508 | 1.191 | 25.478 |
| Reason= Psychiatric disorders | 0.077 | N/C | N/C | 0.069 | 0.047 | 0.1 |
| Reason= Psychiatric disorders * basis= advanced | 0 | N/C | N/C | 0.334 | N/C | N/C |
| year | 1.134 | N/C | N/C | 1.042 | 1.034 | 1.051 |
| Year * basis= advanced | 0.912 | N/C | N/C | 0.923 | 0.905 | 0.941 |
| Year * reason= Dementia | 1.022 | N/C | N/C | 1.049 | 1.018 | 1.08 |
| Year * reason= Dementia * basis= advanced | 0.978 | N/C | N/C | 0.956 | 0.855 | 1.07 |
| Year * reason= Psychiatric disorders | 0.996 | N/C | N/C | 1.013 | 0.988 | 1.04 |
| Year * reason= Psychiatric disorders * basis= advanced | 0.616 | N/C | N/C | 0.002 | N/C | N/C |

## Predicted counts and rates by basis for euthanasia
