# Supplementary material for: Trends in assisted dying among patients with psychiatric disorders and dementia in Belgium: A health registry study
Source: PLoS Med. 2025 Nov 19;22(11):e1004522. doi: 10.1371/journal.pmed.1004522 (PMC12646481; doi:10.1371/journal.pmed.1004522)
Supplement: S6 File — (DOCX) [file pmed.1004522.s006.docx]

# S.6. Zero-inflated negative binomial regression of Reason by Year and expected term of death (three-way interaction)

| Variable | No offset | 95%CI + | 95%CI - | With offset | 95%CI + | 95%CI - |
| --- | --- | --- | --- | --- | --- | --- |
| (Intercept) | 0.035 | 0.030 | 0.041 | 0.000 | 0.000 | 0.000 |
| Age group= 15-29 | 0.056 | 0.032 | 0.098 | 0.032 | 0.015 | 0.068 |
| Age group= 30-39 | 0.236 | 0.176 | 0.315 | 0.235 | 0.159 | 0.348 |
| Age group= 40-49 | 0.486 | 0.401 | 0.589 | 0.449 | 0.360 | 0.561 |
| Age group= 60-69 | 1.402 | 1.223 | 1.608 | 1.950 | 1.648 | 2.308 |
| Age group= 70-79 | 1.553 | 1.355 | 1.780 | 3.177 | 2.586 | 3.903 |
| Age group= 80-89 | 1.568 | 1.364 | 1.803 | 5.694 | 4.474 | 7.248 |
| Age group= 90+ | 0.556 | 0.480 | 0.644 | 12.616 | 9.318 | 17.081 |
| Gender= male | 0.786 | 0.735 | 0.840 | 1.128 | 1.040 | 1.225 |
| Language= NL | 3.692 | 3.455 | 3.945 | 1.945 | 1.721 | 2.197 |
| Reason= Dementia | 0.018 | 0.009 | 0.039 | 0.016 | 0.007 | 0.035 |
| Reason= Dementia * term= Not short term | 16.143 | 6.449 | 40.406 | 16.971 | 6.673 | 43.159 |
| Reason= Psychiatric disorders | 0.007 | 0.002 | 0.023 | 0.006 | 0.002 | 0.021 |
| Reason= Psychiatric disorders * term= Not short term | 125.200 | 36.377 | 430.909 | 146.462 | 41.585 | 515.835 |
| Term= Not short term | 0.073 | 0.062 | 0.086 | 0.068 | 0.056 | 0.083 |
| year | 1.137 | 1.128 | 1.145 | 1.068 | 1.042 | 1.094 |
| Year * reason= Dementia | 0.939 | 0.890 | 0.992 | 0.951 | 0.900 | 1.005 |
| Year * reason= Dementia * term= Not short term | 1.056 | 0.991 | 1.125 | 1.053 | 0.988 | 1.123 |
| Year * reason= Psychiatric disorders | 0.942 | 0.868 | 1.023 | 0.954 | 0.878 | 1.037 |
| Year * reason= Psychiatric disorders * term= Not short term | 1.023 | 0.939 | 1.116 | 1.016 | 0.931 | 1.109 |
| Year * term= Not short term | 1.054 | 1.042 | 1.067 | 1.058 | 1.044 | 1.072 |

## Predicted counts and rates by expected term of death
