# Supplementary material for: Trends in assisted dying among patients with psychiatric disorders and dementia in Belgium: A health registry study
Source: PLoS Med. 2025 Nov 19;22(11):e1004522. doi: 10.1371/journal.pmed.1004522 (PMC12646481; doi:10.1371/journal.pmed.1004522)
Supplement: S7 File — (DOCX) [file pmed.1004522.s007.docx]

# S.7. Zero-inflated negative binomial regression of Reason by Year and type of suffering (three-way interaction)

| Variable | No offset | 95%CI + | 95%CI - | With offset | 95%CI + | 95%CI - |
| --- | --- | --- | --- | --- | --- | --- |
| (Intercept) | 0.036 | 0.029 | 0.044 | 0.000 | 0.000 | 0.000 |
| Age group= 15-29 | 0.032 | 0.025 | 0.042 | 0.025 | 0.019 | 0.034 |
| Age group= 30-39 | 0.100 | 0.084 | 0.119 | 0.107 | 0.089 | 0.129 |
| Age group= 40-49 | 0.304 | 0.264 | 0.349 | 0.315 | 0.271 | 0.367 |
| Age group= 60-69 | 1.978 | 1.760 | 2.224 | 2.366 | 2.093 | 2.675 |
| Age group= 70-79 | 2.297 | 2.049 | 2.574 | 3.878 | 3.433 | 4.382 |
| Age group= 80-89 | 2.264 | 2.017 | 2.541 | 6.765 | 5.969 | 7.668 |
| Age group= 90+ | 0.831 | 0.729 | 0.946 | 12.181 | 10.594 | 14.006 |
| Gender= male | 0.975 | 0.914 | 1.040 | 1.254 | 1.173 | 1.341 |
| Language= NL | 3.392 | 3.165 | 3.636 | 2.108 | 1.960 | 2.266 |
| Reason= Dementia | 0.021 | 0.005 | 0.082 | 0.021 | 0.005 | 0.081 |
| Reason= Dementia * suffering= both | 0.659 | 0.139 | 3.126 | 0.665 | 0.141 | 3.131 |
| Reason= Dementia * suffering= mental | 7.706 | 1.696 | 35.019 | 7.932 | 1.758 | 35.793 |
| Reason= Psychiatric disorders | 0.016 | 0.003 | 0.080 | 0.016 | 0.003 | 0.079 |
| Reason= Psychiatric disorders * suffering= both | 1.379 | 0.242 | 7.857 | 1.392 | 0.246 | 7.864 |
| Reason= Psychiatric disorders * suffering= mental | 39.208 | 7.318 | 210.076 | 40.198 | 7.567 | 213.546 |
| Suffering= both | 2.304 | 1.939 | 2.738 | 2.302 | 1.936 | 2.738 |
| Suffering= mental | 0.247 | 0.189 | 0.323 | 0.244 | 0.187 | 0.319 |
| year | 1.043 | 1.032 | 1.054 | 1.025 | 1.014 | 1.036 |
| Year *reason= Dementia | 0.937 | 0.846 | 1.037 | 0.936 | 0.846 | 1.036 |
| Year * reason= Dementia * suffering= both | 1.092 | 0.976 | 1.222 | 1.091 | 0.976 | 1.220 |
| Year * reason= Dementia * suffering= mental | 1.283 | 1.149 | 1.432 | 1.281 | 1.148 | 1.430 |
| Year * reason= Psychiatric disorders | 0.929 | 0.823 | 1.047 | 0.928 | 0.824 | 1.046 |
| Year * reason= Psychiatric disorders * suffering= both | 1.071 | 0.942 | 1.219 | 1.071 | 0.943 | 1.217 |
| Year * reason= Psychiatric disorders * suffering= mental | 1.252 | 1.104 | 1.420 | 1.252 | 1.105 | 1.419 |
| Year * suffering= both | 1.026 | 1.014 | 1.038 | 1.026 | 1.014 | 1.038 |
| Year * suffering= mental | 0.897 | 0.879 | 0.916 | 0.898 | 0.880 | 0.916 |

## Predicted relative risks and incidence ratios by type of suffering
