# Supplementary material for: Trends in assisted dying among patients with psychiatric disorders and dementia in Belgium: A health registry study
Source: PLoS Med. 2025 Nov 19;22(11):e1004522. doi: 10.1371/journal.pmed.1004522 (PMC12646481; doi:10.1371/journal.pmed.1004522)
Supplement: S8 File — (DOCX) [file pmed.1004522.s008.docx]

# S.8. Zero-inflated negative binomial regression of Reason by Year and place of death (three-way interaction)

| Variable | No offset | 95%CI + | 95%CI - | With offset | 95%CI + | 95%CI - |
| --- | --- | --- | --- | --- | --- | --- |
| (Intercept) | 0.031 | N/C | N/C | 0.000 | 0.000 | 0.000 |
| Age group= 15-29 | 0.043 | N/C | N/C | 0.031 | 0.024 | 0.040 |
| Age group= 30-39 | 0.186 | N/C | N/C | 0.130 | 0.110 | 0.152 |
| Age group= 40-49 | 0.432 | N/C | N/C | 0.345 | 0.303 | 0.393 |
| Age group= 60-69 | 1.530 | N/C | N/C | 2.377 | 2.133 | 2.649 |
| Age group= 70-79 | 1.791 | N/C | N/C | 4.587 | 4.124 | 5.101 |
| Age group= 80-89 | 1.877 | N/C | N/C | 9.423 | 8.460 | 10.497 |
| Age group= 90+ | 0.667 | N/C | N/C | 21.530 | 19.040 | 24.345 |
| Gender= male | 0.811 | N/C | N/C | 1.104 | 1.038 | 1.173 |
| Language= NL | 3.893 | N/C | N/C | 2.089 | 1.956 | 2.232 |
| Place= Hospital | 1.452 | N/C | N/C | 1.474 | 1.217 | 1.787 |
| Place= Nursing home | 0.121 | N/C | N/C | 0.098 | 0.078 | 0.125 |
| Place= Other | 0.053 | N/C | N/C | 0.042 | 0.031 | 0.059 |
| Place= Palliative care | 0.005 | N/C | N/C | 0.003 | 0.002 | 0.005 |
| Reason= Dementia | 0.037 | N/C | N/C | 0.026 | 0.013 | 0.051 |
| Reason= Dementia * Place= Hospital | 0.572 | N/C | N/C | 0.591 | 0.206 | 1.692 |
| Reason= Dementia * Place= Nursing home | 2.793 | N/C | N/C | 3.251 | 0.960 | 11.003 |
| Reason= Dementia * Place= Other | 0.703 | N/C | N/C | 0.796 | 0.060 | 10.590 |
| Reason= Dementia * Place= Palliative care | 0.000 | N/C | N/C | 0.000 | 0.000 | #NUM! |
| Reason= Psychiatric disorders | 0.093 | N/C | N/C | 0.085 | 0.049 | 0.150 |
| Reason= Psychiatric disorders * Place= Hospital | 0.458 | N/C | N/C | 0.445 | 0.182 | 1.087 |
| Reason= Psychiatric disorders * Place= Nursing home | 2.446 | N/C | N/C | 2.401 | 0.834 | 6.908 |
| Reason= Psychiatric disorders * Place= Other | 1.057 | N/C | N/C | 0.921 | 0.214 | 3.967 |
| Reason= Psychiatric disorders * Place= Palliative care | 0.000 | N/C | N/C | 0.000 | 0.000 | N/C |
| Year | 1.152 | N/C | N/C | 1.070 | 1.058 | 1.082 |
| Year * Place= Hospital | 0.964 | N/C | N/C | 0.962 | 0.948 | 0.975 |
| Year * Place= Nursing home | 1.060 | N/C | N/C | 1.061 | 1.044 | 1.078 |
| Year * Place= Other | 0.980 | N/C | N/C | 0.994 | 0.973 | 1.017 |
| Year * Place= Palliative care | 1.144 | N/C | N/C | 1.184 | 1.152 | 1.218 |
| Year * reason= Dementia | 1.018 | N/C | N/C | 1.041 | 0.996 | 1.088 |
| Year * reason= Dementia * Place= Hospital | 1.004 | N/C | N/C | 1.005 | 0.938 | 1.077 |
| Year * reason= Dementia * Place= Nursing home | 0.958 | N/C | N/C | 0.960 | 0.889 | 1.036 |
| Year * reason= Dementia * Place= Other | 1.066 | N/C | N/C | 1.059 | 0.908 | 1.236 |
| Year * reason= Dementia * Place= Palliative care | 18325.132 | N/C | N/C | 20316.171 | N/C | N/C |
| Year * reason= Psychiatric disorders | 1.005 | N/C | N/C | 1.021 | 0.982 | 1.061 |
| Year * reason= Psychiatric disorders * Place= Hospital | 0.993 | N/C | N/C | 0.990 | 0.930 | 1.054 |
| Year * reason= Psychiatric disorders * Place= Nursing home | 0.913 | N/C | N/C | 0.915 | 0.852 | 0.983 |
| Year * reason= Psychiatric disorders * Place= Other | 1.089 | N/C | N/C | 1.092 | 0.997 | 1.196 |
| Year * reason= Psychiatric disorders * Place= Palliative care | 3.370 | N/C | N/C | 3.343 | 0.556 | 20.093 |
