# Supplementary material for: Trends in assisted dying among patients with psychiatric disorders and dementia in Belgium: A health registry study
Source: PLoS Med. 2025 Nov 19;22(11):e1004522. doi: 10.1371/journal.pmed.1004522 (PMC12646481; doi:10.1371/journal.pmed.1004522)
Supplement: S9 File — (DOCX) [file pmed.1004522.s009.docx]

# S.9. Zero-inflated negative binomial regression, predicted counts and rates of Reason by Year (factor) (two-way interaction)
