# Supplementary material for: Trends in assisted dying among patients with psychiatric disorders and dementia in Belgium: A health registry study
Source: PLoS Med. 2025 Nov 19;22(11):e1004522. doi: 10.1371/journal.pmed.1004522 (PMC12646481; doi:10.1371/journal.pmed.1004522)
Supplement: Attachment — Submitted filename: PLOSONE - review.docx [file pmed.1004522.s011.docx]

**Peer Review of the Manuscript: "Addressing the Slippery Slope Argument: Trends in Euthanasia Among Patients with Psychiatric Disorders and Dementia in Belgium, 2002–2023"**

**General Assessment**

The manuscript presents an important and timely investigation into trends in euthanasia among patients with psychiatric disorders and dementia in Belgium. The authors employ a robust **time-series Poisson regression model** using administrative data from the Federal Commission for the Control and Evaluation of Euthanasia (FCCEE). The study’s strengths lie in its empirical approach to assessing the "slippery slope" argument and its comparative focus on demographic and regional trends.

While the article is **methodologically sound and well-structured**, there are several issues requiring attention, particularly regarding **terminological consistency**, **interpretation of findings**, and **framing of ethical implications**.

**Major Concerns:**

**1. Inconsistent Use of Terminology: Euthanasia vs. Assisted Suicide**

One of the main weaknesses of the manuscript is the **lack of consistency in distinguishing between "euthanasia" and "assisted suicide."** These terms are often used interchangeably, yet they represent **distinct concepts**. In Belgium, euthanasia is legally defined as the deliberate act of ending a patient's life at their explicit request, performed by a physician. Assisted suicide, on the other hand, involves providing the means for a patient to end their own life. The authors should clarify their usage throughout the paper and ensure they **distinguish between these terms** when discussing Belgian law versus international comparisons.

- **Suggested Revision:** Define both terms clearly in the introduction and maintain consistency throughout the text. If the dataset does not include assisted suicide cases, the term should not be used at all.

**2. The Interpretation of the "Slippery Slope" Argument**

The paper does an excellent job addressing the "slippery slope" claim empirically. However, its **discussion lacks nuance** in addressing why concerns about an expanding scope of euthanasia persist in public debates.

- **Missed Perspective:** While the data does not support a dramatic increase in euthanasia cases, the concern over **ethical precedent** (i.e., what conditions justify euthanasia beyond physical terminal illnesses) is not adequately acknowledged.
- **Suggested Addition:** Include a brief discussion of the broader ethical concerns beyond statistical trends, particularly issues raised by disability rights groups and psychiatric professionals.

**3. Gender and Regional Analysis Requires More Interpretation**

The paper presents interesting findings regarding gender and regional trends but does not provide sufficient theoretical explanations for these differences.

**Issues to Address:**

- Why are women overrepresented in psychiatric euthanasia? Are there societal, cultural, or healthcare-related factors that explain this?
- Why does the French-speaking region have a steeper increase? Are there differences in healthcare accessibility or cultural attitudes?
- **Suggested Addition:** A brief discussion integrating sociocultural and healthcare system factors influencing these trends.

**4. Data Interpretation and Ethical Considerations**

The study effectively highlights the **importance of safeguards in euthanasia legislation**. However, the **policy implications remain vague**, especially regarding:

- **Potential Overreach in Conclusions:** The authors argue that Belgium’s experience **"debunks the slippery slope argument"**, but the data **only show that overall numbers remain low and stable**. The fact that euthanasia for dementia is increasing (albeit modestly) suggests **some level of policy evolution** over time.
- **Suggested Revision:** The authors should **rephrase their conclusions** to reflect that while a "slippery slope" is not evident in numerical trends, ethical debates about the scope of euthanasia remain legitimate.
- **How Belgium ensures mental competence in psychiatric euthanasia cases**
- **What safeguards exist for vulnerable populations in dementia-related euthanasia**
- **Why Belgium’s model differs from countries that prohibit psychiatric euthanasia (e.g., Germany, UK)**
- **Recommendation**: The authors should briefly discuss **international perspectives on psychiatric euthanasia** and **potential safeguards needed in Belgium moving forward**.

**Minor Issues & Suggestions**

**1. Formatting and Grammar**

- There are **several minor typos**, such as "methos" instead of "methods."
- Some sentences are overly complex and could be streamlined for clarity.

**2. Statistical Methods Section**

- The Poisson regression model is appropriate for count data, but it would be helpful to **briefly explain why a negative binomial model was not considered**, as euthanasia cases may exhibit overdispersion.

**3. Clarity in Data Representation**

- Figures and tables are useful, but **some trends should be more explicitly explained in the text** to avoid misinterpretation.

#### 4. **Abstract Improvements**

- Clarify that euthanasia trends differ for **psychiatric disorders vs. dementia**.

This study provides a valuable contribution to the **debate on euthanasia for psychiatric and cognitive disorders**, using a **robust dataset and statistical approach**. However, **terminological precision and a more balanced interpretation** of findings are necessary before publication.

I look forward to seeing a revised version that **incorporates these key improvements**.
