# Supplementary material for: Trends in assisted dying among patients with psychiatric disorders and dementia in Belgium: A health registry study
Source: PLoS Med. 2025 Nov 19;22(11):e1004522. doi: 10.1371/journal.pmed.1004522 (PMC12646481; doi:10.1371/journal.pmed.1004522)
Supplement: Attachment — Submitted filename: Answers.docx [file pmed.1004522.s012.docx]

Table of Contents

[Reviewer #1	4](#_Toc197615692)

[Reviewer #2:	6](#_Toc197615693)

[Reviewer #3:	10](#_Toc197615694)

[Reviewer #4:	13](#_Toc197615695)

# Reviewer #1

"Addressing the Slippery Slope Argument: Trends in Euthanasia Among Patients with Psychiatric Disorders and Dementia in Belgium, 2002-2023" analyzed data on all reported cases of euthanasia in Belgium over about two decades via time-series Poisson regression, to determine whether euthanasia trends for psychiatric disorders and dementia indicate a possible "slippery slope". It was found that while euthanasia rates for psychiatric disorders followed similar trends as for other types of euthanasia, dementia had a slight over-increase only partly explained by demographic change. Nevertheless, it was concluded that there was no evidence of significant misuse of euthanasia regulations allowing for non-terminal psychiatric conditions.

While informative, some issues might be considered:

1. Some additional details could be included in the Introduction, to establish the context. In particular,

a) How is euthanasia administered, and what is the typical waiting time between the initial request(s), approval, and administration?

Answer: Thank you for this important request for clarification. We do describe the method of intravenous administration, which typically involves a sedative, followed by a barbiturate, and, if necessary, a neuromuscular blocking agent. We also clarify that Belgian law distinguishes between deaths expected in the near term and those not expected within a foreseeable timeframe, which determines whether the standard or reinforced procedural pathway applies.

b) Would withdrawal of care count as euthanasia for terminally-ill patients?

Answer: In Belgian law, euthanasia is strictly defined as the act of intentionally ending a patient’s life by a third party at the patient’s explicit request. By contrast, the withdrawal or withholding of life-sustaining treatment (such as artificial hydration, nutrition, or ventilation) is considered part of standard medical decision-making at the end of life and is not classified as euthanasia. Euthanasia requires a formal written request and compliance with strict legal safeguards, while treatment withdrawal is guided by medical practice and patient consent, particularly in cases where continued treatment is deemed futile or disproportionate. In such situations, the relevant legal framework is the Law on Patients’ Rights.

c) Is there any specific definition of terminal illness, in terms of expected remaining lifespan and/or suffering?

Answer: Thank you for this important question. Belgian law does not define terminal illness in terms of a fixed expected lifespan. Instead, it differentiates between situations where death is expected “in the near term” and those where it is not. The law interprets “near term” as death anticipated within weeks or months. This distinction determines the procedural pathway: when death is not expected in the near term (as with many psychiatric or neurodegenerative conditions), a reinforced procedure applies, which includes additional medical consultations and a mandatory one-month waiting period. This clarification is provided in the manuscript.

2. In the Data subsection, it is stated that "The dataset contains aggregated count data stratified by multiple demographic, temporal, and contextual factors. Each row represents a unique combination of attributes, including year, language, gender, age, age group, location, and reason for the case... The dataset is structured as a rectangular table, with each row corresponding to a unique combination of predictor variables, ensuring that all possible combinations are represented, including those with zero counts". This description might be clarified - does it mean that the dataset has 127 unique combinations of (the 7) attributes?

Answer: Thank you for this important question. To keep the data & method section clear and tidy, we have added information on the number of lines at the start of the “Analyses” section as follow (p.14): “The dataset contains 126,720 lines in total, with each line reporting the number of euthanasia cases (including zero counts). These lines represent combinations of population characteristics (gender [2] × language [2] × age group [8] = 32) and euthanasia characteristics (reason [3] × place of death [5] × type of suffering [3] × term of death [2] × basis of euthanasia [2] = 180) across 22 years.”

3. In the Reasons for euthanasia subsesction, it might be clarified as to whether the "multimorbidity" category applies whenever a patient would otherwise fall into two or more of the other categories (possibly including psychiatric disorders/dementia).

Answer: This is a very important comment, thank you. We have clarified this in the text as follow (p.12): . “It should be noted that cases involving multimorbidity may include dementia or psychiatric disorders, but always together with a terminal illness that would, alone, qualify for euthanasia; the FCCEE data, however, do not contain homogeneous ready-to-code information on multimorbidity.”

4. Further, it may be appropriate to consider the baseline prevalence of the various medical conditions, over the time period.

Answer: Thank you also for this important point. Our primary objective was to match euthanasia data by conditions with the prevalence of these conditions observed within the full Belgian population. Alas, the nomenclature used by the euthanasia committee does not match the categories of the INAMI/RIZIV (Institut national d'assurance maladie-invalidité; Rijksinstituut voor ziekte- en invaliditeitsverzekering). Specifically, we did not find aggregate data on multimorbidity and could not proceed further. This will be a next step once we have access to the full information within the multimorbidity category.

5. In the Population offset subsection, assumptions for language are stated. They might be further justified by relevant citations or otherwise, if possible.

Answer: the 10% estimates is based on previous demographic figures. We have included a reference in the text as follow (p.13): “This 10 percent figure is an estimate of the Dutch-speaking population residing in Brussels. In 2001, 8.4 percent of Brussels residents were born in Flanders, and this proportion has slightly increased over time [43]”.

# Reviewer #2:

General Assessment

The manuscript presents an important and timely investigation into trends in euthanasia among patients with psychiatric disorders and dementia in Belgium. The authors employ a robust **time-series Poisson regression model** using administrative data from the Federal Commission for the Control and Evaluation of Euthanasia (FCCEE). The study’s strengths lie in its empirical approach to assessing the "slippery slope" argument and its comparative focus on demographic and regional trends.

While the article is **methodologically sound and well-structured**, there are several issues requiring attention, particularly regarding **terminological consistency**, **interpretation of findings**, and **framing of ethical implications**.

Major Concerns:

1. Inconsistent Use of Terminology: Euthanasia vs. Assisted Suicide

One of the main weaknesses of the manuscript is the lack of consistency in distinguishing between "euthanasia" and "assisted suicide." These terms are often used interchangeably, yet they represent distinct concepts. In Belgium, euthanasia is legally defined as the deliberate act of ending a patient's life at their explicit request, performed by a physician. Assisted suicide, on the other hand, involves providing the means for a patient to end their own life. The authors should clarify their usage throughout the paper and ensure they distinguish between these terms when discussing Belgian law versus international comparisons.

- Suggested Revision: Define both terms clearly in the introduction and maintain consistency throughout the text. If the dataset does not include assisted suicide cases, the term should not be used at all.

Answser: Thank you for this helpful observation. We agree that euthanasia and assisted suicide are conceptually and legally distinct. However, our manuscript focuses exclusively on cases reported to the Belgian Federal Commission for the Evaluation and Control of Euthanasia (CFCEE), which, under Belgian law, pertains specifically to euthanasia—defined as a life-ending act performed by a physician at the explicit request of the patient.

Although assisted suicide is not explicitly provided for in Belgian legislation, the CFCEE has noted that in very rare cases (0.6% of reported euthanasia cases), the administration of a barbiturate syrup by the patient—under continuous supervision and responsibility of a physician—may resemble what is internationally referred to as “medical assistance in dying” or “assisted suicide.” Both the CFCEE and the National Council of the Belgian Medical Order (opinion of 22 March 2003) consider this practice to fall within the legal framework of euthanasia, provided that all legal safeguards are met and that the physician remains continuously present and ready to intervene throughout the process.

Given that the CFCEE treats such cases within the scope of euthanasia, we have opted not to separate them analytically. Furthermore, as our article does not engage in international legal comparisons—an important topic that would merit a separate discussion—we chose to use the term “euthanasia” throughout in accordance with Belgian legal and institutional usage.

2. The Interpretation of the "Slippery Slope" Argument

The paper does an excellent job addressing the "slippery slope" claim empirically. However, its discussion lacks nuance in addressing why concerns about an expanding scope of euthanasia persist in public debates.

- Missed Perspective: While the data does not support a dramatic increase in euthanasia cases, the concern over ethical precedent (i.e., what conditions justify euthanasia beyond physical terminal illnesses) is not adequately acknowledged.
- Suggested Addition: Include a brief discussion of the broader ethical concerns beyond statistical trends, particularly issues raised by disability rights groups and psychiatric professionals.

Answer: Thank you for this thoughtful suggestion. We agree that concerns about the ethical implications of an expanding scope of euthanasia—particularly beyond terminal physical illness—persist in public and professional debates, even when not reflected in the quantitative trends. In the Background section, we have aimed to acknowledge this tension by discussing high-profile legal cases (e.g., Tine Nys), institutional positions such as the 2017 opinion of the Belgian Advisory Committee on Bioethics, and the broader discourse surrounding psychiatric suffering and decision-making capacity. These elements help contextualize why the “slippery slope” argument remains salient in ethical debates, even in the absence of a substantial rise in cases.

3. Gender and Regional Analysis Requires More Interpretation

The paper presents interesting findings regarding gender and regional trends but does not provide sufficient theoretical explanations for these differences.

Issues to Address:

- Why are women overrepresented in psychiatric euthanasia? Are there societal, cultural, or healthcare-related factors that explain this?

Answer: This appears to be a consistent finding across countries where assisted dying or euthanasia is permitted for psychiatric disorders. One possible explanation is the higher prevalence – or higher diagnosis rate – of psychiatric disorders among women. Unfortunately, we do not have access to data on psychiatric disorder rates in the Belgian population disaggregated by year, gender, and age group. As a result, we are unable to assess whether the higher proportion of female cases reflects underlying prevalence patterns. Any extrapolation would therefore be speculative and based on assumptions rather than empirical evidence, which we prefer to avoid.

- Why does the French-speaking region have a steeper increase? Are there differences in healthcare accessibility or cultural attitudes?
- Suggested Addition: A brief discussion integrating sociocultural and healthcare system factors influencing these trends.

Answer: Thank you for raising this important point. We have attempted to clarify our findings in the text. Overall, euthanasia cases are more frequent in the Flemish region. However, our study observes a growing number of cases in the French-speaking region, with the south of the country gradually catching up to the north. As you mention, there are two potential explanations: cultural attitudes – among both patients and the medical community – and health care access. Our study does not directly address these factors, as we do not examine attitudes towards euthanasia (which could be investigated using, for example, ESS data), and the language variable we use – limited to two categories – does not permit a proper analysis of geographical disparities in health care access. This is an area we aim to explore further; we are currently collecting communal-level data to better understand access disparities across sub-populations. Since our paper does not adequately address either aspect with the available data, we chose not to elaborate further on the regional divide.

4. Data Interpretation and Ethical Considerations

The study effectively highlights the **importance of safeguards in euthanasia legislation**. However, the **policy implications remain vague**, especially regarding:

- Potential Overreach in Conclusions: The authors argue that Belgium’s experience "debunks the slippery slope argument", but the data only show that overall numbers remain low and stable. The fact that euthanasia for dementia is increasing (albeit modestly) suggests some level of policy evolution over time.
- Suggested Revision: The authors should rephrase their conclusions to reflect that while a "slippery slope" is not evident in numerical trends, ethical debates about the scope of euthanasia remain legitimate.

Answer: Thank you very much for both comments. We have fully re-written the discussion section and clarify these two points in pages 20 to 23.

- **How Belgium ensures mental competence in psychiatric euthanasia cases**
- **What safeguards exist for vulnerable populations in dementia-related euthanasia**

**Answer: We have fully restructured the background section and include the detail on the safeguards for both psychiatric and euthanasia cases in pages 5 to 11.**

- **Why Belgium’s model differs from countries that prohibit psychiatric euthanasia (e.g., Germany, UK)**
- **Recommendation**: The authors should briefly discuss **international perspectives on psychiatric euthanasia** and **potential safeguards needed in Belgium moving forward**.

Answer: Thank you for this insightful comment. We agree that a broader international perspective on psychiatric euthanasia is important and that comparative studies can offer valuable context. However, as our study is based solely on Belgian data, incorporating detailed comparisons with countries like Germany or the UK—where psychiatric euthanasia is prohibited—would be beyond the scope of our current analysis. While we do touch on non-Belgian regulations in the background and briefly mention developments in France and the UK in the discussion, a more in-depth comparative analysis would require dedicated data from these countries. We are cautious that attempting to draw broader comparisons without such data may weaken the specificity and rigor of our findings. That said, we acknowledge the importance of safeguards in the Belgian context and have included a brief note on this in the discussion.

Minor Issues & Suggestions

1. Formatting and Grammar

- There are several minor typos, such as "methos" instead of "methods."
- Some sentences are overly complex and could be streamlined for clarity.

Answer: Thank you for pointing out these typos.

2. Statistical Methods Section

- The Poisson regression model is appropriate for count data, but it would be helpful to briefly explain why a negative binomial model was not considered, as euthanasia cases may exhibit overdispersion.

Answer: Thank you very much for this helpful comment. We initially used Poisson regression because this is the most straightforward method. However, we did some sensitivity check and decided to re-run our models using a zero-inflated binomial model (with and without offset). We explain this in the methods section on page 14 to 16. Results are not different except the trend for psychiatric disorders that is slightly increasing with the negative binomial whilst it was somehow more stable in the Poisson regression model (this is a very minor difference). We briefly discuss these differences in the limitation section.

3. Clarity in Data Representation

- Figures and tables are useful, but some trends should be more explicitly explained in the text to avoid misinterpretation.

Answer: Thank you very much for this comment. We initially wanted to keep the paper as short as possible but, as PLOS Medicine does not limit the number of characters with have improved the results section and include descriptions of the main table and the two figures.

#### 4. Abstract Improvements

- Clarify that euthanasia trends differ for psychiatric disorders vs. dementia.

Answer: We have modified the abstract to include the following: “Although slightly increasing, euthanasia for psychiatric disorders followed trends similar to the other types of euthanasia (count=1.00 [95%CI: 0.98; 1.03] – rate=1.02 [95%CI: 0.99; 1.04]), while euthanasia for dementia showed an over-increase (count= 1.03 [95%CI: 1.00; 1.06] – rate= 1.04 [95%CI: 1.01;1.07]).”

This study provides a valuable contribution to the **debate on euthanasia for psychiatric and cognitive disorders**, using a **robust dataset and statistical approach**. However, **terminological precision and a more balanced interpretation** of findings are necessary before publication.

I look forward to seeing a revised version that **incorporates these key improvements**.

# Reviewer #3:

Summary:

This is a population-based register-analysis of aggregated data to investigate the alleged "slippery slope"-phenomena in euthanasia. Data from 2002, the year of initiating euthanasia in Belgium, to 2023 is used and incidence rates and -ratios are calculated to answer questions around the time trend of euthanasia for different underlaying diagnoses, with a particular focus on dementia and psychiatric illnesses. This type of scientific contribution is sorely needed in the field, the aim is admirable, and the analysis are ambitious but there are some clarifications needed, both in analytic approach and in presentation.

Major comments:

1.      To me, the text is not objective, but biased in favour of euthanasia, in the choice of word and phrases, please consider revision. (A read-through by a "critical friend" might help)

Answer: Thank you very much for your comment—this is an important point. From the outset of our work on this topic, we have observed that much of the existing literature appears to take a critical stance toward euthanasia, often framed through ethical arguments. While this is understandable given the sensitivity of the topic, we feel that neutrality is not always consistently applied across the debate. That said, we want to clarify that our intention was not to present a partisan position. We do not hold strong personal views on the matter; rather, our aim was to examine the available data—something that is relatively rare in this field—and to present findings that, based on the evidence, do not appear to indicate anything alarming. We regret if this was interpreted as advocacy. In response to feedback, we have revised the manuscript to add further nuance, particularly in the background and discussion sections, in order to better reflect the complexity of the issue and ensure the tone is as balanced as possible.

2.      Please clarify the aim in relation to the "slippery slope". Since euthanasia was allowed from the beginning for non-terminal illnesses, including psychiatric ones, in Belgium it is unclear to me what slippery slope that is investigated in the paper. Is it a question of inferring what the more ethically troublesome parts of euthanasia are, and looking into those diagnoses?

Answer: Thank you for this comment. Our argument initially has two components. First, the "slippery slope" argument suggests that granting a right – such as access to euthanasia for terminal illness – will inevitably lead to its extension to non-terminal conditions. The Belgian case provides a counterpoint to this assumption, as both types of cases (terminal and non-terminal) have been permitted from the outset. This is a perspective that is not often addressed in the existing literature. Second, the concern is that legalizing euthanasia may lead to a sharp increase in cases, including among patients who fall outside the intended scope of the law. Our findings suggest that, over a 22-year period, cases of euthanasia for psychological distress have remained relatively stable. However, there has been an increase in cases involving dementia. Nevertheless, we recognize that the slippery slope framework may not be the most appropriate lens for addressing our specific research questions. As a result, we have reduced its emphasis in the paper and narrowed the focus of our analysis accordingly.

3.      I acknowledge your take on statistical significance, it is a standpoint but not mine, in my mind Belgium is not the whole world, and 2002-2023 it not all time, therefore, if we wish to generalize the findings to other times and/or places, we need the statistical significance tests. They describe the uncertainty of the data and contribute important information. Especially if one would like to compare IRRs, to say that one is more pronounced than the other, but both estimates are based on very small numbers I think the CIs are very important indeed.

Answer: Thank you for this comment. We have now added 95% confidence intervals (CIs) to the paper, as we agree they contribute valuable information for interpreting the robustness of the results. We would like to clarify, however, that statistical significance testing is traditionally used to assess whether an estimate derived from a sample is likely to reflect the true value in the overall population. In our case, the data are not a sample but comprise the full population of reported euthanasia cases in Belgium between 2002 and 2023. Therefore, confidence intervals and significance levels are not required to generalize from a sample to a population—they are, in this context, used more to reflect model stability and estimation precision. We also agree that Belgium is not representative of all jurisdictions that allow assisted dying, and we do not attempt to generalize our findings beyond the Belgian context. In that sense, confidence intervals should not be interpreted as a means to support extrapolation to other countries or time periods. However, we recognize that including 95% CIs is useful for two key reasons. First, they provide a transparent indication of the precision of our estimates, especially when interpreting rate ratios across groups. For example, in some of the more complex models—such as the three-way interactions examining advanced requests—confidence intervals are not presented due to convergence issues arising from very small case numbers, particularly since advance euthanasia requests for dementia are not currently legal in Belgium. Second, providing confidence intervals may be helpful for future systematic reviews or comparative studies, allowing researchers to incorporate our findings into broader international analyses. We therefore thank the reviewer for this valuable suggestion.

4.      I find the analyses difficult to interpret (I note that the authors do not interpret their estimates in words either very much either). Since I am not a statistician, I suggest to the editor for your paper to go to statistical review, but I think there have to be more work done on the analysis, as this is not the customary way to do either IRR or interactions. I would suggest considering a structure like this:

Table 2: Incidence rates (IR) i.e. number of cases/100 000 person-years, for different subtypes of euthanasia, and the total, per year. Be clear in the heading of the table what is shown, the table (with heading and notes) should be self-explanatory.

Table 3: Incidence rate ratios (IRR). Give the estimate by year (e.g. IRR=1.03, there is 3% increase in the probability to die from euthanasia each year). Then, test the statistical significance of the interaction terms that you choose (e.g. There is a statistically significant interaction between year and reason for euthanasia, p=0.03). Then make dummy variables, perhaps grouping the years into categories (e.g. IRR for each year with dementia as reason: 1.02, IRR for each year with other reason: 1.03). (I do not at all require your paper to follow the above exactly, it is intended as a help to describe how an interaction analysis can be presented, I am sure there are many ways, but you current one does not work).

5.      What does the first model, that calculate the "occurrence" (frequency?) of euthanasia, contribute with? Does this model take person-time at risk into account at all? Also, I do not find much value in the graphs unfortunately. What does "predicted" mean? Estimated?

Answer: Thank you for this comment and we agree that the estimates in the original version of the paper are hard to interpret. We have clarified how they should be interested in the methods section as follow (pp.14-15): “In Model 1 (without offset), the exponentiated coefficients represent multiplicative changes in the expected number of euthanasia cases (count) associated with a one-unit increase in the predictor variables. For example, if the exponentiated coefficient for year is 1.05, this suggests that each additional year is associated with a 5% increase in the expected euthanasia count, assuming all other variables remain constant. Similarly, categorical predictors (e.g., gender or age-groups) compare the expected counts between groups. Importantly, this model treats the outcome purely as a count, without accounting for differences in population size. In Model 2 (with offset), the exponentiated coefficients instead reflect changes in the rate of euthanasia per unit of the offset variable (i.e., sub-groups population size). Here, a coefficient of 1.05 for year would mean that each additional year corresponds to a 5% increase in the euthanasia rate (number of cases relative to the offset), holding other variables constant. This model is more appropriate when the underlying population size influence the observed counts and to isolate the effects of predictors on the rate rather than the raw count. “

6.      Interactions are notoriously easy to preform statistically but hard to interpret, please consider excluding the three-way-interactions, (a hint of this is that you yourself state that it is hard to interpret, follow your gut in this case).

Answer: Thank you for your comment. You are absolutely right—while 3-way interactions are relatively straightforward to estimate, they are often challenging to interpret, especially for readers who may already find single-factor effects complex. Nonetheless, we found this approach necessary to explore dynamics such as changes over time by gender or region. To enhance interpretability, we have provided predicted values from the key models in figure 2 and offer a detailed explanation of these results on pages 19-20. We hope that these visualisations and descriptions offer a clearer and more accessible interpretation than the raw coefficients in Table 2.

Minor comments:

7.      Be clear in the year of study start, is it 2002 or 2003?

Answer: This was a mistake, thank you very much for pointing it out. The study starts in 2002. We found similar trends when restricting to 2003 onwards and decided to keep the full data.

8.      "Informed consent" on p.5 can't possibly be the correct term for requesting euthansia?

Answer: This comes from the French word ‘consentement’ and is indeed the term used by the law. (i.e., the patient make a request and the medical practitioner address their consent).

9.      There are some missing references, e.g. Statbel and R.

10.     The incidence rate is called a "prevalence rate" in the method.

Answer: Thank you for pointing this out. We use the terms of counts and rates in this second version to keep the paper simple.

11.     Twelve medical conditions that are categorized into 7, that are then categorized again into 3: I am not certain that the description of the 7 categories adds value (Methods, p.8).

Answer: Thank you very much for pointing this out. Previous studies grouped the 12 conditions into seven but this did not make sense in our paper. We have removed the seven conditions information from the methods section.

I reserve the right to add additional comments, primarily on wording, if the authors choose to submit a revised version of the manuscript.

# Reviewer #4:

This is a scholarly paper in that it uses sophisticated statistical techniques to provide some detailed analysis of complicated data. However, I think you have undermined the academic standing of the paper by your focus (in the paper's title and in its content) on the slippery slope argument. You could publish your results without any reference to the slippery slope argument, in which case the paper would provide something interesting for the research community to ponder. Unfortunately, the focus on the slippery slope argument means that the whole endeavour is wasted.

For a start, if you are going to use your data to attack or undermine the slippery slope argument, you should start with the best account of that argument that you cane find. Instead, you provide only a cursory definition of the argument. Indeed, right from the start (p. 4) you assert that the argument is "fallacious". If the argument is so obviously fallacious, why bother to say anything further. But the argument is not obviously "fallacious". Indeed, in the best hands, there are two aspects to the argument (it's perhaps better to refer to "arguments" in the plural), an empirical aspect and a logical aspect (see Keown (2018) 'Euthanasia, Ethics and Public Policy' pp. 67-89). You might say that you are obviously only applying your analysis to the empirical argument. But that would leave the other aspects of the argument(s) intact, so you would not have achieved your objective and your conclusions would be wrong. In any case, although empirical data can be relevant to the argument, in the end the argument is a normative one which needs to be addressed at a conceptual level not at a statistical one.

There is a very basic point to be made about how the Belgian law demonstrates a slippery slope. The law was passed in 2002 to allow voluntary active euthanasia. Then, in 2014 the Euthanasia Act was amended to allow euthanasia for minors. In so doing, Belgium slid some way down a slippery slope. Full stop. We don't need any further empirical analysis. The eligibility criteria have been extended in keeping with the slippery slope argument(s).

But there are lots of other ways in which this slippage can be demonstrated: the move from serious and incurable disorders to multiple disorders, the stretching of the notion of suffering, the inclusion of physician-assisted suicide, and so on. Keown (2018) discusses all of this in some detail (pp.298-325) in a chapter entitled 'Belgium's lack of effective control'. Proper academic discussion of the issue would surely need to include some mention of Keown (2018), where there are two chapter devoted purely to the experience in Belgium.

Moreover, I would also have expected some discussion of the  book 'Euthanasia and Assisted Suicide: Lessons from Belgium' edited by Jones, Gastmans and MacKellar and published in 2017. One of the things that these works highlight is the number of cases of euthanasia which are not reported, which would also be relevant to a discussion of the possibility of slippery slopes.

There is another point. Whenever euthanasia or assisted suicide are legalised in a country, the rates of "assisted dying" gradually rise. Those against "assisted dying" point to this increasing prevalence as a sign that it is getting out of control; those in favour argue that one would expect the prevalence to increase and suggest it shows that "assisted dying" is acceptable. But, of course, the numbers don't on their own tell us anything, because we don't know what the normal prevalence should be; and, moreover, there is no way of knowing. What is normal here requires an evaluative decision. We need a completely different type of research to say whether or not the prevalence is (normatively) high or low. The same sort of difficulty faces your research. But in any case, the evidence that there is some sort of slippery slope in Belgium is apparent and is not simply based on opinion.

Answer: Thank you for your comment and your time on this paper. We have reformed our paper to focus only on the slippery slope from an empirical perspective, not from an ethical point of view.

Nevertheless, we should mention that this paper is about cases of euthanasia for psychiatric disorders and dementia. The change in regulation in 2014 allowing people aged 16 and over to request for euthanasia under strict guidelines does not apply to psychiatric disorders and dementia. Therefore, this change of regulations could be used to fuel the slippery slope argument on these specific aspects.

Regarding the book you mentioned, 'Euthanasia and Assisted Suicide: Lessons from Belgium' edited by Jones, Gastmans and MacKellar (2017) , we have decided not to mention it because this is propaganda from the Opus Dei. Etienne Montero who wrote several pages in this book is now an official priest from the Opus Dei (but was a professor back in 2017).

General editorial requests:

(Note: not all will apply to your paper, but please check each item carefully)

*       We ask every co-author listed on the manuscript to fill in a contributing author statement, making sure to declare all competing interests. If any of the co-authors have not filled in the statement, we will remind them to do so when the paper is revised. If all statements are not completed in a timely fashion this could hold up the re-review process. If new competing interests are declared later in the revision process, this may also hold up the submission. Should there be a problem getting one of your co-authors to fill in a statement we will be in contact. Please do not add or remove authors without first discussing this with the handling editor. You can see our competing interests policy here: <http://journals.plos.org/plosmedicine/s/competing-interests>.

Answer: Done

*       Please upload any figures associated with your paper as individual TIF or EPS files with 300dpi resolution at resubmission; please read our figure guidelines for more information on our requirements: <http://journals.plos.org/plosmedicine/s/figures>. While revising your submission, please upload your figure files to the PACE digital diagnostic tool, <https://pacev2.apexcovantage.com/>. PACE helps ensure that figures meet PLOS requirements. To use PACE, you must first register as a user. Then, login and navigate to the UPLOAD tab, where you will find detailed instructions on how to use the tool. If you encounter any issues or have any questions when using PACE, please email us at [PLOSMedicine@plos.org](mailto:PLOSMedicine@plos.org).

*       Please ensure that the paper adheres to the PLOS Data Availability Policy (see <http://journals.plos.org/plosmedicine/s/data-availability>), which requires that all data underlying the study's findings be provided in a repository or as Supporting Information. For data residing with a third party, authors are required to provide instructions with contact information (web or email address) for obtaining the data. Please note that a study author cannot be the contact person for the data. PLOSjournals do not allow statements supported by "data not shown" or "unpublished results." For such statements, authors must provide supporting data or cite public sources that include it.

*       We expect all researchers with submissions to PLOS in which author-generated code underpins the findings in the manuscript to make all author-generated code available without restrictions upon publication of the work. In cases where code is central to the manuscript, we may require the code to be made available as a condition of publication. Authors are responsible for ensuring that the code is reusable and well documented. Please make any custom code available, either as part of your data deposition or as a supplementary file. Please add a sentence to your data availability statement regarding any code used in the study, e.g. "The code used in the analysis is available from Github [URL] and archived in Zenodo [DOI link]" Please review our guidelines at <https://journals.plos.org/plosmedicine/s/materials-software-and-code-sharing> and ensure that your code is shared in a way that follows best practice and facilitates reproducibility and reuse. Because Github depositions can

be readily changed or deleted, we encourage you to make a permanent DOI'd copy (e.g. in Zenodo) and provide the URL.

FORMATTING - GENERAL

*       Abstract: Please structure your abstract using the PLOS Medicine headings (Background, Methods and Findings, Conclusions). Please combine the Methods and Findings sections into one section.

Answer: Done.

*       At this stage, we ask that you include a short, non-technical Author Summary of your research to make findings accessible to a wide audience that includes both scientists and non-scientists. The Author Summary should immediately follow the Abstract in your revised manuscript. This text is subject to editorial change and should be distinct from the scientific abstract. Ideally each sub-heading should contain 2-3 single sentence, concise bullet points containing the most salient points from your study. In the final bullet point of 'What Do These Findings Mean?', please include the main limitations of the study in non-technical language. Please see our author guidelines for more information: <https://journals.plos.org/plosmedicine/s/revising-your-manuscript#loc-author-summary>.

*       Please express the main results with 95% CIs as well as p values. When reporting p values please report as p<0.001 and where higher as the exact p value p=0.002, for example. Throughout, suggest reporting statistical information as follows to improve clarity for the reader "22% (95% CI [13%,28%]; p</=)". Please be sure to define all numerical values at first use.

Answer: We have included the 95%CI in this second version of the article for transparency but the use of 95%CI and the interpretation the p-value make no sense in this study because we use total population data and not sample data.

*       Please include page numbers and line numbers in the manuscript file. Use continuous line numbers (do not restart the numbering on each page).

Answer: Done

*       Please cite the reference numbers in square brackets. Citations should precede punctuation.

Answer: Done

FIGURES AND TABLES

*       Please provide titles and legends for all figures and tables (including those in Supporting Information files).

*       Please define all abbreviations used in each figure/table (including those in Supporting Information files).

*       Please consider avoiding the use of red and green in order to make your figure more accessible to those with color blindness.

SUPPLEMENTARY MATERIAL

*       Please note that supplementary material will be posted as supplied by the authors. Therefore, please amend it according to the relevant comments outlined here.

*       Please cite your Supporting Information as outlined here: <https://journals.plos.org/plosmedicine/s/supporting-information>

REFERENCES

*       PLOS uses the numbered citation (citation-sequence) method and first six authors, et al.

*       Please ensure that journal name abbreviations match those found in the National Center for Biotechnology Information (NCBI) databases (<http://www.ncbi.nlm.nih.gov/nlmcatalog/journals>), and are appropriately formatted and capitalised.

*       Where website addresses are cited, please include the complete URL and specify the date of access (e.g. [accessed: 12/06/2023]).

*       Please also see <https://journals.plos.org/plosmedicine/s/submission-guidelines#loc-references> for further details on reference formatting.

OBSERVATIONAL STUDIES

*       Abstract: Please include the study design, population and setting, number of participants, years during which the study took place (enrollment and follow up), length of follow up, and main outcome measures.

*       Please ensure that the study is reported according to the STROBE (or appropriate STOBE extension) guideline (available from: <https://www.equator-network.org/reporting-guidelines/strobe>) and include the completed STROBE (or STROBE extension) checklist as Supporting Information. Please add the following statement, or similar, to the Methods: "This study is reported as per the Strengthening the Reporting of Observational Studies in Epidemiology (STROBE) guideline (S1 Checklist)." When completing the checklist, please use section and paragraph numbers, rather than page numbers.

Answer: Done

*       [FOR POPULATION HEALTH/REGISTRY STUDIES] Please ensure that the study is reported according to the RECORD guideline (available from <https://www.record-statement.org>) and include the completed checklist as Supporting Information. Please add the following statement, or similar, to the Methods: "This study is reported as per the Reporting of Studies Conducted using Observational Routinely-Collected Data (RECORD) guideline (S1 Checklist)." When completing the checklist, please use section and paragraph numbers, rather than page numbers.

*       [FOR POPULATION HEALTH ESTIMATES] Please ensure that the study is reported according to the GATHER statement (available from <https://www.equator-network.org/reporting-guidelines/gather-statement>) and include the completed checklist as Supporting Information. Please add the following statement, or similar, to the Methods: "This study is reported as per the Guidelines for Accurate and Transparent Health Estimates Reporting (GATHER) statement (S1 Checklist)." When completing the checklist, please use section and paragraph numbers, rather than page numbers.

*       For all observational studies, in the manuscript text, please indicate: (1) the specific hypotheses you intended to test, (2) the analytical methods by which you planned to test them, (3) the analyses you actually performed, and (4) when reported analyses differ from those that were planned, transparent explanations for differences that affect the reliability of the study's results. If a reported analysis was performed based on an interesting but unanticipated pattern in the data, please be clear that the analysis was data driven.

*       Please state in the Methods section whether the study had a prospective protocol or analysis plan. If a prospective analysis plan (from your funding proposal, IRB or other ethics committee submission, study protocol, or other planning document written before analyzing the data) was used in designing the study, please include the relevant document(s) with your revised manuscript as a Supporting Information file to be published alongside your study and cite it in the Methods section. A legend for this file should be included at the end of your manuscript. If no such document exists, please make sure that the Methods section transparently describes when analyses were planned, and when/why any data-driven changes to analyses took place. Changes in the analysis, including those made in response to peer review comments, should be identified as such in the Methods section of the paper, with rationale.
