# Supplementary material for: Trends in assisted dying among patients with psychiatric disorders and dementia in Belgium: A health registry study
Source: PLoS Med. 2025 Nov 19;22(11):e1004522. doi: 10.1371/journal.pmed.1004522 (PMC12646481; doi:10.1371/journal.pmed.1004522)
Supplement: Attachment — Submitted filename: Reviews-2.docx [file pmed.1004522.s013.docx]

The 31^st^ of August 2025

Dear Dr De Bruijn,

We would like to thank you and the reviewers for the opportunity to revise and resubmit our manuscript, “Trends in Assisted Dying Among Patients with Psychiatric Disorders and Dementia in Belgium” (PMEDICINE-D-24-04457R2). We are grateful for the time and expertise invested by the reviewers and for the constructive suggestions that have helped us improve the clarity, neutrality, and methodological transparency of our work.

In response to the specific editorial requests, we carefully revised the manuscript sentence by sentence to ensure a neutral tone, with particular attention to the Abstract and Discussion. We removed all causal language in the Abstract, restructured its conclusion, and eliminated references to court cases in the Introduction. The Introduction has been shortened in line with the word count request, and the Discussion has been trimmed to better focus on the study’s findings.

We also removed references to the slippery slope argument throughout the manuscript. However, given the centrality of this concept to broader debates on assisted dying, we felt that completely omitting it from the Discussion would risk leaving the paper incomplete. As a compromise, we retained a final paragraph that acknowledges the debate but reframes the issue in a more neutral and empirical way, clarifying that our study does not directly test ethical claims but contributes methodological rigor to ongoing discussions.

We would like to extend our sincere thanks to Reviewer 1 and Reviewer 2, whose thoughtful and constructive comments greatly contributed to strengthening the manuscript. Reviewer 1’s methodological feedback was particularly helpful in refining our statistical framework, and Reviewer 2’s detailed suggestions on terminology, policy framing, and language refinement led to significant improvements in both clarity and balance.

We also thank Reviewer 3 for their engagement with our work. At the same time, we felt that some of the feedback was expressed in a manner that at times came across as less neutral than expected in the peer review process. For example, the suggestion that our writing reflects a “lack of scientific integrity” and the rhetorical comparison questioning whether one would use the term “catching up” in relation to cancer incidence could be perceived as unnecessarily confrontational rather than focused on scientific critique. We nevertheless took these comments seriously and used them as an opportunity to further improve the paper. In particular, we revised the Abstract conclusion to remove any implication of overreach, clarified the role of 95% confidence intervals, and rephrased passages in the Discussion to more explicitly emphasize the limits of our analysis and scope. We also provided further clarification on why the first descriptive model was retained, i.e., to illustrate how trends appear when demographic change is not accounted for, and how this can contribute to misleading interpretations in prior literature.

We hope that these revisions have addressed the concerns of the editorial team and reviewers. We remain committed to ensuring the manuscript reflects an accurate, balanced, and empirically grounded contribution to the debate on assisted dying.

We thank you again for your time and the reviewers’ engagement, and we look forward to your further consideration of our revised manuscript.

Sincerely,

The corresponding author

Dear Dr Wels,

Many thanks for submitting your manuscript "Trends in Assisted Dying Among Patients with Psychiatric Disorders and Dementia in Belgium" (PMEDICINE-D-24-04457R2) to PLOS Medicine. The paper has been reviewed by subject experts and a statistician; their comments are included below and can also be accessed here: ********

As you will see, the reviewers thought the manuscript has improved but they still have serious concern about bias. After discussing the paper with the editorial team and an academic editor with relevant expertise, we agreed to offer you one more round of revision to address this issue. We plan to send the revised paper to some or all of the original reviewers, and we cannot provide any guarantees at this stage regarding publication.

Specifically we would like you to address the following:
1) Revise the language sentence by sentence. This applies to the whole manuscript, but the Abstract and Discussion in particular. 
Specific points:
-remove the causal language on line 36 of the abstract.
-remove line 42-44 of the abstract and re-write this.

- **We have removed the causal language on line 36 and improved the conclusion of the abstract.**

2) Please remove any use of the slippery slope argument completely, and instead just focus on the examination of the trends in euthanasia cases. This can be along the lines of “Legislation for ADE was adopted in 2002 in Belgium permitting euthanasia for terminal illness and for non-terminal illnesses such as XYZ. In this study we examined trends in euthanasia cases involving patients psychiatric disorders, dementia and terminal illness from 200X-20XX”

- We removed the references to the slipper slope arguments in the article and focusing on the examination of the trends in euthanasia cases, as requested. We have included a paragraph in the discussion section that mention the term but mentioning all the limitations discussed by the reviewers.

3) Please remove the court case discussion in the introduction, instead it would be fine to state it’s a contentions issue. Court cases could be mentioned in the discussion only.
4) Please shorten your introduction to 750 words.

- **We have shortened the introduction and removed the court case for the article.**

5) Please trim the discussion, as this is overly long currently.

- **Except for the last paragraph, we have trimmed the discussion section by focusing on the results study results.**

In addition to these revisions, you will need to complete some formatting changes, which you will receive in a follow up email. A member of our team will be in touch with a set of requests shortly.

When you upload your revision, please include a point-by-point response that addresses all of the reviewer and editorial points, indicating the changes made in the manuscript and either an excerpt of the revised text or the location (eg: page and line number) where each change can be found. Please also be sure to check the general editorial comments at the end of this letter and include these in your point-by-point response. When you resubmit your paper, please include a clean version of the paper as the main article file and a version with changes tracked as a marked-up manuscript. It may also be helpful to check the guidelines for revised papers at http://journals.plos.org/plosmedicine/s/revising-your-manuscript for any that apply to your paper.

We ask that you submit your revision by Jul 21 2025 11:59PM. However, if this deadline is not feasible, please contact me by email, and we can discuss a suitable alternative.

Don't hesitate to contact me directly with any questions (sbruijn@plos.org). 

Best regards, 
Suzanne 

Suzanne De Bruijn, PhD 
Associate Editor
PLOS Medicine
sbruijn@plos.org

-----------------------------------------------------------
Comments from the academic editor:

Please ensure that they text is neutral. I suggest to examine every sentence to consider whether the text is justified, and whether there is any implicit bias still there. Maybe imagine you are someone very much against assisted suicide, and read the manuscript from their perspective.

-----------------------------------------------------------
Comments from the reviewers: 

Reviewer #1: We thank the authors for addressing our previous comments, and the other reviewers for improving the statistical analyses.

- **Thank you very much for your very helpful comments that contributed to improve the methodological framework of our study.**

Reviewer #2: I sincerely thank the authors for carefully addressing the reviewers' comments. The revisions have significantly improved the clarity and quality of the manuscript. 
There still remains minor issues to be addressed:
1. Terminology: Euthanasia vs. Assisted Suicide
The clarification provided in the methods and background sections regarding the exclusive use of "euthanasia" in line with Belgian legal definitions is well explained. However, I recommend adding a brief footnote at the first mention of the term "euthanasia" to inform international readers that in Belgium, this may include rare cases of supervised self-administration that are still legally classified as euthanasia.

- **We have added the following sentence “Euthanasia is typically administered intravenously but rare cases rare cases of supervised self-administration exit (0.3 percent of all cases in 2022-23) within the description of the Belgian regulation is the methods section. (This section was moved out from the background section to keep it short, as requested by the editors).**

Concerning terminologia, i point that assisted dying is commonly used as an umbrella term , including euthanasia and assisted suicide. For this reason, I suggests that authors to use only the term assisted dying and not assisted dying and euthanasia.

- **Thank you for this comment. You are absolutely correct that the term assisted dying is the commonly used umbrella term. We have added a footnote at the end of our first sentence that states the following:**

**“For the purposes of this paper, "assisted dying" serves as an umbrella term for assisted dying, assisted suicide and euthanasia. The term "euthanasia" is used when discussing Belgium, in alignment with its statutory language”.**

2. Interpretation of the Slippery Slope Argument
The revised manuscript commendably distinguishes between the empirical and normative dimensions of the "slippery slope" argument.
Suggestion:
I recommend including an explicit statement in the discussion acknowledging that while the empirical data do not support a slippery slope in numerical terms, ethical concerns about the broadening of eligibility criteria remain valid and deserve further comparative research.
3. Gender and Regional Trends
The authors have appropriately acknowledged data limitations and refrained from speculation regarding gender and regional disparities.
Further Recommendation:
Consider moving part of this discussion into the limitations section, where it may be more visible. 
4. Policy and Limitations
The discussion section has been usefully restructured.
Additional Suggestion:
The authors may wish to state more explicitly that while dementia-related euthanasia remains infrequent, the upward trend over time is noteworthy and warrants monitoring.
5. Minor issues 
- The shift to a zero-inflated negative binomial model is appropriate and well justified.
- Including 95% confidence intervals greatly improves transparency.
- Some defensive phrasing remains, especially regarding critical literature (e.g., references to the 2017 edited volume). I encourage the authors to maintain a more neutral and academic tone throughout.
Conclusion
Overall, this is a strong and timely study. With a few remaining refinements to language and emphasis, I believe the manuscript will be ready for publication.

- **Thank you very much for your very helpful comments, these helped to improve the paper substantially.**

**As requested by reviewers #3 and the Editor(s), it was requested that we remove our reference to the slippery slope argument. We discuss this further in reviewers #3 comments.**

Reviewer #3: I acknowledge that the authors have invested into the revision of the manuscript, and improvements has been achieved, however there is still significant issues remaining. 

1. Formulations in the text still puts into question the neutrality of the authors in terms of euthanasia as a concept. Would one for example use the phrase "catching up" if cancer incidence of one region were approaching another region with higher incidence? This is not easily fixed with further revisions, since the text is a sign of the problem, rather than the problem itself.

- **Thank you for this comment. The paragraph you are referring to is not about cancer but about euthanasia for dementia or psychiatric disorders. We tackle the conditions propensities issue for dementia. One main driver of dementia propensities is population ageing and the study adjust for population change at regional legal. It is still possible that the rise of cases of euthanasia for psychiatric disorders in French-speaking Belgium is explained by increasing number of cases, and this is a limitation as psychiatric disorders propensities are not available by regions and year. Whilst this is a methodological limitation, we do not believe that this is a matter of neutrality.**

Also, in abstract conclusion is stated "…without evidence of significant misuse…" the misuse, or lack of it, has not been investigated, only time trends. The fact that this is the conclusion after another reviewer pointed out that the conclusions were overreaching is troublesome.

- **Thank you for this important comment. You are correct to say that the study did not address this aspect in particular and we have revised the abstract conclusion.**

2. There is a lack of scientific integrity shown in the discrepancy between the answer to me regarding 95%CIs: "We have now added 95% confidence intervals (CIs) to the paper, as we agree they contribute valuable information for interpreting the robustness of the results." Vs the text in the manuscript: "the 95% CIs are unnecessary and do not provide meaningful insights." (row 412 in tracked changes version).

- **We apologize for the confusion, the “the 95% CIs are unnecessary and do not provide meaningful insights” sentence was supposed to be deleted and we forgot to do so in the previous version. Thank you for pointing this out.**

In addition, the authors state that they do not wish to extrapolate the findings outside of Belgium. In the second paragraph of the Discussion (rows 569-584) the authors reason around the situation in UK and France, concluding "The Belgian experience, however, addresses this concern." I don't mind this, I fact I think this is why we do research at all: to be able to extrapolate into the unknown, (the etiology of cancer tumours not yet developed e.g.), but the fact the authors don't see that this is what they doing is concerning.

- **We thank the reviewer for this comment but would like to clarify that the previous reviewer’s request was as follows**: “**Why Belgium’s model differs from countries that prohibit psychiatric euthanasia (e.g., Germany, UK). Recommendation**: **The authors should briefly discuss international perspectives on psychiatric euthanasia** **and** **potential safeguards needed in Belgium moving forward.” As explained in our previous answer, there is no room in this article to compare countries. Furthermore, as reviewers prevent us from addressing the slippery slope because of the limited nature of the data, we find contradictory that they want use to discuss other countries whilst we do not analyze non-Belgian data. When we mention the UK and France in the conclusion, we are talking about countries that are currently debating the implementation of some assisted dying regulations and currently have no regulation, and this is not a comparison of the Belgian regulation with other regulations. Our previous reply read as follows:**
  - **“We agree that a broader international perspective on psychiatric euthanasia is important and that comparative studies can offer valuable context. However, as our study is based solely on Belgian data, incorporating detailed comparisons with countries like Germany or the UK—where psychiatric euthanasia is prohibited—would be beyond the scope of our current analysis. While we do touch on non-Belgian regulations in the background and briefly mention developments in France and the UK in the discussion, a more in-depth comparative analysis would require dedicated data from these countries.”**

**However, we have rephrased the conclusion to mention that our study only focuses on Belgian data. This reads as follows: “In the Belgian context, this was not a concern as the Belgian law has allowed euthanasia for non-terminal and non-physical illnesses under specific safeguards, enforced by medical practitioners and monitored by the Federal Commission for the Control and Evaluation of Euthanasia from the outset.” (lines 394-396).**

**We are in the process of collecting international data for a broader international comparison of ongoing legislation to better address these issues based on actual data and we hope the reviewers will be contacted at a point to review this future project.**

3. I appreciate the decreased focus and usage of the term/concept "slippery slope", however, it is still present (e.g. in the conclusion of the Abstract). This is from the revised abstract: "Assisted dying and euthanasia (ADE) for patients with psychiatric disorders or dementia have increased in countries where the practice is legal. These cases are often cited in arguments against ADE legislation, as they are perceived to contribute to a potential slippery slope in case numbers." The fact that the case numbers increase, or not, does not tell us if that is good (good uptake of new legislation, the opportunity of new treatment reaches all part of the population) or bad (misuse, lax definition of eligible patients), that is an ethical interpretation, not empirical. It is built into the "slippery slope"-argument that an increase is bad, but the increase itself does not carry any value when not coupled with an ethical standpoint. Which is why I would wish the authors really kept only to analyses of the data and conclusion that can be drawn from these.

- **Thank you for this important comment. This is not our main point regarding the slippery slope argument. Our concern is that discussions about the potential slippery slope often rely on flawed descriptive statistics that fail to account for population change and thus present a biased view that is rarely questioned. The issue is not only ethical but also empirical: how can an ethical debate be built on quantitative information that is inaccurate? While our article does not directly address the ethical aspects of the so-called slippery slope (as we stated in our previous version), it does challenge certain misconceptions. Although the notion is widely used by policymakers and professional bodies, we regret that the slippery slope argument is often supported only by unsubstantiated—and frequently flawed—studies. To comply with your requests and the editor’s instructions, we have removed references to the slippery slope argument from the main body of the article. It is now clearer that the article does not directly challenge the notion. However, we have included a concluding paragraph that acknowledges the issue and explains why more rigorous data analyses could inform ethical debates:**

**“We conclude with a reflection on how empirical evidence is mobilized in public debate. Among the arguments that have shaped discussions on euthanasia is the so-called slippery slope, raised both in parliamentary debates [44] and within professional medical bodies [45]. This argument has two dimensions: an ethical one, expressing the concern that permitting euthanasia in limited cases (e.g., terminal illness) may gradually lead to less justified applications; and an empirical one, questioning whether, over time, safeguards are effectively maintained or whether practices extend to more controversial cases, such as non-terminal conditions or psychiatric disorders [46] . In the literature, the ethical dimension of the slippery slope has received much more attention than the empirical one. Where the empirical dimension is addressed, it is usually based on descriptive trends—often not adjusted for demographic change—that are commonly interpreted as evidence of an expansion of practices [8,12]. Whilst we acknowledge that empirical data cannot resolve ethical and normative questions, our findings add to the debate by showing that euthanasia for psychiatric and cognitive conditions has been legally permissible in Belgium from the outset of the law and has not substantially increased over time. This highlights the importance of methodological rigor, along with the development of diverse methodological approaches, to provide a factual basis for public debate on end-of-life issues and their inherent complexities.” (lines 319-335).**

4. In my first review I asked "What does the first model, that calculate the "occurrence" (frequency?) of euthanasia, contribute with?" this was not answered but the manuscript now states "Importantly, this model treats the outcome purely as a count, without accounting for differences in population size." This would seem to indicate that Model 1 adds no value to the manuscript and should be removed. (Or, an explanation in the manuscript of how the interpretation of model 1 helps the final conclusion is needed) 
(This remind me of papers of my own where we started analysis somewhere, moved on in our thinking and did not until late realize that the first attempts were superfluous in the manuscript, even if they were instrumental for our moving forwards in analytical stage)

- **Thank you for this comment as this is an important point and we carefully thought about removing non-population adjusted analyses. One of the points made by the paper is that previous research and reports examining euthanasia trends in Belgium are misleading because they do not account for population change. This leads to over-estimate euthanasia trends and fuel ethical arguments (that we do not discuss any more in the body of the paper). For transparency, it is important to show this effect in the first figure and explain the difference in the text. We hope that this would also promote a better use of the descriptive data. To clarify this, we have improved our first research question (R.Q.1). This now reads as follows: “R.Q.1 How do trends in euthanasia cases involving psychiatric conditions and cognitive disorders compare with those for other reasons, such as terminal illness, and how does this comparison change when demographic composition is taken into account?”.**
