# Supplementary material for: Trends in assisted dying among patients with psychiatric disorders and dementia in Belgium: A health registry study
Source: PLoS Med. 2025 Nov 19;22(11):e1004522. doi: 10.1371/journal.pmed.1004522 (PMC12646481; doi:10.1371/journal.pmed.1004522)
Supplement: Attachment — Submitted filename: Review 3. PLOS one. 2025.docx [file pmed.1004522.s014.docx]

**Context and Overview**

This review builds upon my previous feedback as Reviewer 2 and reflects a reassessment of the third revised version of the manuscript. I appreciate the authors’ responsiveness and their efforts to engage constructively with reviewer concerns. This is a timely, methodologically sound study that provides valuable empirical data on euthanasia trends for non-terminal conditions in Belgium — a subject of high ethical, legal, and clinical relevance.

The authors have meaningfully addressed earlier concerns regarding statistical modeling, transparency, and tone. However, a small number of remaining issues — particularly related to normative framing and engagement with relevant literature — still warrant refinement before final acceptance.

**Strengths of the Revised Manuscript**

- The statistical framework (zero-inflated negative binomial regression) is well-justified, with clear communication of offset use and confidence intervals.
- The tone has been moderated in many places, with improved clarity in the abstract and discussion.
- Terminology has been clarified, with useful footnotes distinguishing “assisted dying” and “euthanasia” in the Belgian legal context.
- The slippery slope framing is now more cautiously positioned, with a clear distinction between ethical and empirical interpretations.

**Remaining Areas for Improvement**

**A. Normative Framing and Language**

Despite clear improvements, certain phrases remain normatively suggestive, subtly implying that increases or regional convergence in euthanasia for psychiatric/dementia conditions represent progress or improved access. Given the ethical and policy stakes, such language must be avoided.

**Suggested Edits to Improve Neutrality:**

| **Current Phrase** | **Suggested Alternative** | **Rationale** |
| --- | --- | --- |
| “catching up” | “increasing at a faster rate” / “reducing historical differences” | “Catching up” implies progress |
| “harmonization” | “convergence in rates” | Avoids normative value of “uniformity” |
| “adjustment across sub-populations” | “shifts in distribution” or “changes across sub-populations” | Neutral alternative |
| “The Belgian experience addresses this concern.” | “Belgian data offer contextual insights into these concerns.” | Avoids implying resolution of ethical or policy debates |
| “not a concern” (in conclusion) | “no observed increase in frequency based on reported data” | Avoids policy-level conclusions |
| “seems to become more common” | “increased” or “cases have risen over time” | More empirical; avoids conjecture |
| “differences have narrowed, suggesting harmonization” | “regional differences have declined over time” | Removes implied endorsement |

**B. Literature and Debate Engagement**

**1. Omission of Qualitative and Clinical Ethical Literature**

The manuscript does not engage with the substantial qualitative research on assisted dying for psychiatric disorders, especially regarding decision-making capacity, physician uncertainty, or patient ambivalence. These perspectives are essential for contextualizing quantitative trends and understanding the ethical implications of ADE expansion.

**Suggestion**: Acknowledge this gap in the limitations section:

“This study focuses on administrative trends and does not address clinical, psychological, or qualitative dimensions of ADE for psychiatric conditions, such as assessments of capacity or decisional ambivalence. These are crucial areas for future research.”

**2. Underrepresentation of Ethical Diversity**

While the paper attempts to neutralize tone, there is limited engagement with opposing normative frameworks. For example, principled concerns about whether patients with psychiatric suffering can ever make a truly autonomous request for euthanasia are absent, despite their prominence in international debates.

**Suggestion**: In the discussion, include a line such as:

“Our analysis does not resolve ongoing ethical disagreements regarding capacity, suffering, and vulnerability in psychiatric ADE, which remain the subject of principled debate in bioethics and law.”

**3. International Policy Context Still Overstated**

The claim that “the Belgian experience addresses this concern” (re: slippery slope) overstates what the data can prove and implies normative generalizability. Ethical and political contexts differ significantly between Belgium, France, the UK, and elsewhere.

**Suggestion**: Replace with:

“While the Belgian data provide longitudinal insights, caution is warranted in applying these findings to other national contexts where legal frameworks, safeguards, and public attitudes toward assisted dying differ markedly.”

**Final Recommendation**

I commend the authors for the substantial improvements made since R1. This version is very close to being publication ready. With the incorporation of the above adjustments — particularly around normative phrasing, acknowledging ethical diversity, and clarifying limitations — the paper will stand as a rigorous and valuable contribution to the ongoing global debate on assisted dying for psychiatric and cognitive conditions. These are relatively modest changes in wording and framing but important ones, given the ethical sensitivity of the subject matter and the visibility of this paper in future policy discussions. Once these revisions are addressed, I support publication.
