# Supplementary material for: Trends in assisted dying among patients with psychiatric disorders and dementia in Belgium: A health registry study
Source: PLoS Med. 2025 Nov 19;22(11):e1004522. doi: 10.1371/journal.pmed.1004522 (PMC12646481; doi:10.1371/journal.pmed.1004522)
Supplement: Attachment — Submitted filename: Response to reviewers.docx [file pmed.1004522.s015.docx]

London,

The 8^th^ of October 2025

Dear Dr De Bruijn,

We are very happy that this paper has been accepted for publication in PLOS Medicine. We wish to extend our sincere thanks to the editor and reviewers for their thorough and constructive feedback, which has significantly strengthened our manuscript.

We have implemented the vast majority of the requested changes. In the few instances where we suggested a slight alternative phrasing (noted in our point-by-point response), it was to ensure absolute factual accuracy regarding the Belgian legal context or the statistical interpretation of our models.

We are particularly grateful to the editor for the detailed guidance on maintaining a neutral and objective tone throughout the manuscript. This was a challenging aspect of writing on such a topic, and the editorial input was helpful in achieving this.

We also thank Reviewer #2 for their final positive assessment and for the nuanced suggestions on language, which we have incorporated to further enhance the paper's neutrality.

Best wishes,

Jacques Wels

**Point-by-point response**

Requests from Editors:

* Please use the active voice throughout the manuscript.

* Please remove any use of the phrase 'uncontrolled increase'

- Done

* Please move the ‘limitations’ section to the discussion.

- Done

Specific textual requests:

ABSTRACT

Line 20-24 (background): Please change to:

“Assisted dying and euthanasia (ADE) laws make a distinction between ADE for terminal illnesses and non-terminal illnesses such as psychiatric disorders or dementia. ADE for patients with psychiatric disorders or dementia have increased in countries where the practice is legal. In this study, we examine trends in euthanasia cases involving patients with these conditions in Belgium, between 2002 and 2023.

- This would not be fully correct as Belgium does not directly distinguish between terminal and non-terminal illnesses but only one whether the person is expected to die in the near future (defined by the FCCEE by a 12 months period).
- We have also made a mistake in mentioning ‘countries’ as some jurisdictions allow euthanasia within countries (i.e. US States).
- We suggest the following: “Assisted dying and euthanasia (ADE) for patients with psychiatric disorders or dementia have increased in jurisdictions where the practice is legal. In this study, we examine trends in euthanasia cases involving patients with these conditions in Belgium, where the law makes a distinction based on whether a patient's death is not expected in the foreseeable future (>12 months) – a common situation in cases of dementia or psychiatric disorders.”

Line 32-35 (methods and findings). Please change to:

“Although slightly increasing, euthanasia for psychiatric disorders followed trends similar to the other types of euthanasia (count=1.00 [95%CI: 0.98; 1.03] – rate=1.02 [95%CI: 0.99; 1.04]), while euthanasia cases for dementia increased faster than other types of dementia (count= 1.03 [95%CI: 1.00; 1.06] – rate= 1.04 [95%CI: 36 1.01;1.07]).

- Done

Line 41: please change ‘discussion’ into ‘Conclusion’.

- Done

Line 41- 46 (Conclusion): Please change to:

“In Belgium, between 2002 and 2023, there are distinct trends for euthanasia for non-terminal illnesses. Euthanasia for psychiatric disorders followed similar trends as euthanasia for terminal illnesses, whereas euthanasia cases involving cognitive conditions increased at a faster rate. Furthermore, there were gender and regional differences, which diminished over time.”

- Done

BACKGROUND

Line 58-60: change to:

“The discourse primarily focuses on two categories of conditions where death is not expected within 12 months: dementia and other degenerative disorders, and severe psychiatric illnesses.”

- We have changed this and move the section the reinforced procedure in the methods/reason for euthanasia section: “In other words, in cases of euthanasia when death is not expected in the foreseeable future (defined by the FCCEE as a period of 12 months or more) – such as for psychiatric disorders or dementia or cognitive conditions –, a reinforced procedure applies. In this case, a second physician (i.e., independent psychiatrist or specialist in the condition prompting the euthanasia) should be consulted, and a mandatory waiting period applies [1]. Euthanasia is typically administered intravenously, but rare cases of supervised self-administration exist (0.3 percent of all cases in 2022-23 [26]).”

Line 65-67: remove ‘actual’ , remove ‘leaving little room for empirical evidence’.

- Done

Line 67: remove ‘Similarly’.

- Done

Line 71: change ‘demand’ to ‘numbers of cases’

- Done

Line 73-76: change to:

“The observed increase in cases fuel concerns about a gradual expansion of eligibility criteria, particularly leading to concerns regarding vulnerable patients. These concerns are used as an argument against implementing ADE for non-terminal illness in other jurisdictions and as an argument in favour of introducing stricter safeguards.”

- Done

Line 80-82:change to:

“the idea that eligibility criteria in ADE regulations are gradually expanded, or that vulnerable populations are at greater risk is not substantiated by empirical studies.”

- Done

Line 83 remove ‘notably’ before focused.

- Done

RESULTS:

Line 258-261: Please change to:

“Over the selected period, psychiatric disorders and dementia represent 1.27% and 0.92% of all cases, respectively, and followed similar trends to the overall number of cases.”

- Done

Line 264: Besides psychiatric conditions and cognitive disorders, are there any other non-terminal illnesses reason for euthanasia? If not, please change ‘with those for other reasons’ to ‘with those for terminal illnesses’.

- This would not be correct because, among euthanasia cases for other reasons, some are not for terminal illness (i.e., some multimorbidity cases are sometimes not considered as terminal illness).

Line 288-293: Change to:

“the difference between predicted rates with and without adjustment for population composition and change suggests that population change may partially underlie changes observed in euthanasia prevalence. Furthermore, we observe that the rate of increase in euthanasia cases is similar for terminal illnesses and psychiatric disorders, but higher for dementia cases.”

- To keep the nuance that the ‘other’ cases may also involve non-terminal illness: we wrote the following: “The difference between predicted rates with and without adjustment for population composition and change suggests that population change may partially underlie changes observed in euthanasia prevalence. Furthermore, the rate of change in relatively similar when comparing euthanasia for psychiatric disorders to the other types of causes but the increase is sharper for dementia.”

Line 311-313: Change to:

‘However, over time the rate of increase is becoming larger in Wallonia-Brussels compared with Flanders, indicating that the observed difference between these two regions is diminishing.’

- We wrote the following to keep the interpretation of the estimates accurate: “However, the rate of change is lower for the Flanders compared to Wallonia-Brussels over the selected period (Year x Dementia x NL, rate: 0.963 [95%CI: 0.897; 1.034] – Year x Psychiatric disorders x NL , rate: 0.865 [95%CI: 0.777; 0.962].”

Line 322-324: what is meant here? ‘other’ reasons involving mental suffering? Or all euthanasia cases for ‘other’ (which sounds unlikely?). Please clarify.

- We are sorry about this, the sentence was not clear. We clarified for “Mental suffering was higher in cases of euthanasia for dementia and psychiatric disorders compared to non-psychiatric/dementia cases (dementia × mental suffering, rate = 7.932 [95%CI: 1.75; 35]) – Psychiatric disorders x mental suffering, rate= 40.198 [95%CI: 7.567; 213]).”

Line 328-329: Please remove this sentence, as there is no statistical significance for these findings.

- We have changed the sentence for the following because significance is not relevant when working with full population data but the point is important: “Data indicate a potential increase of euthanasia for dementia and psychiatric disorders in palliative care but low counts lead to convergence issue and this trend should be taken with caution.”

Line 341-347: change to:

“These cases have been more prevalent in the Dutch-speaking community than the French-speaking community, although this regional difference has diminished over time. Furthermore, we observed that most of the increase in psychiatric and dementia-related euthanasia occurred in the French-speaking community.”

- Done

Line 350-353: Please remove this paragraph. Especially the last sentence ‘Overall, much of the increase in euthanasia cases is driven by short-term death expectations, rather than long-term ones.’ as this statement is not directly linked to the goal of the study.

- We have deleted this sentence.

Line 360-363: Please remove these 2 sentences, as it seems overly political, and can be perceived as bias.

- Done

Line 367: remove ‘proactively’

- Done

Line 370-375: Please reconsider this paragraph. ‘methods used to analyse ADE figures….’ As this seems to criticize other studies, rather than highlighting a limitation of the current study.

- Done

DISCUSSION:

Line 385-396: change to:

“Recent debates on the implementation of assisted dying in the united Kingdom and France have highlighted the distinction between euthanasia for terminal- and non-terminal causes, with both these countries considering implementation of euthanasia for terminal-illnesses only, excluding patients with psychiatric disorders or degenerative conditions.

In contrast, Belgium euthanasia law has allowed euthanasia for non-terminal and non-physical illnesses from its inception in 2002, allowing us to compare rates in euthanasia for terminal illnesses, psychiatric disorders, and dementia.

- Done

Line 397-: remove ‘dramatic increase’. Suggested text:

“In 2023, psychiatric disorders and dementia accounted for 1.4% and 1.2% of all cases, respectively. We observed a significant, but still modest, increase in euthanasia for dementia. Furthermore, we observed that differences across genders and linguistic regions have narrowed over time.

- We rephrased the paragraph following your suggestion. This now reads as: “Our study shows a significant, but still modest, increase in euthanasia for dementia. Furthermore, differences across genders and linguistic regions have narrowed over time. Trends show gradual implementation, with a steady yearly rate of change for psychiatric conditions and a slightly sharper but still modest increase for dementia. During the first five years following the implementation of the regulation, cases of euthanasia for psychiatric disorders and dementia remained rare, indicating that medical practitioners were reluctant at first to practice euthanasia on patients suffering from cognitive or psychiatric conditions. Differences in access across genders and linguistic regions have narrowed over time. In 2023, psychiatric disorders and dementia accounted for 1.4% and 1.2% of all cases, respectively.”

Line 414-418: Please consider removing this paragraph; without data on changes in quantity/quality of palliative care units, this adds little to the discussion.

- Done

Line 419-435; please change the last paragraph to:

“Concerns have been raised, both in parliamentary debates [39] and within professional medical bodies, that permitting euthanasia in limited cases (e.g., terminal illness) may gradually lead to less justified applications, and whether safeguards are effectively maintained when euthanasia is extended to non-terminal conditions or psychiatric disorders [41] . Whilst we acknowledge that empirical data cannot resolve ethical and normative questions, our findings demonstrate that euthanasia for psychiatric and cognitive conditions, which has been legally permissible in Belgium since 2002, has not substantially increased between 2002 and 2023. These findings can add to the discussion of end-of-life issues and their inherent complexities.”

- We have changed it. Thank you for rephrasing this paragraph.

GENERAL EDITORIAL REQUESTS

* Thank you for providing an Author summary. Can you please include this within the main manuscript, directly following the Abstract. In the final bullet point of ‘What Do These Findings Mean?’ Please include the main limitations of the study in non-technical language.

* Please confirm that your title complies with to PLOS Medicine's style. Your title must be nondeclarative and not a question. It should begin with main concept if possible. "Effect of" should be used only if causality can be inferred, i.e., for an RCT. Please place the study design ("A randomized controlled trial," "A retrospective study," "A modelling study," etc.) in the subtitle (ie, after a colon).

* Please confirm that your abstract complies with our requirements, including format (three sections: Background, Methods and Findings, and Conclusions) and providing all the information relevant to this study type <https://eur01.safelinks.protection.outlook.com/?url=https%3A%2F%2Fjournals.plos.org%2Fplosmedicine%2Fs%2Fsubmission-guidelines%23loc-abstract&data=05%7C02%7Cw.jacques%40ucl.ac.uk%7C5d33d293d5944b68577108de0659a288%7C1faf88fea9984c5b93c9210a11d9a5c2%7C0%7C0%7C638955179507173667%7CUnknown%7CTWFpbGZsb3d8eyJFbXB0eU1hcGkiOnRydWUsIlYiOiIwLjAuMDAwMCIsIlAiOiJXaW4zMiIsIkFOIjoiTWFpbCIsIldUIjoyfQ%3D%3D%7C0%7C%7C%7C&sdata=90xwE4dbLHrcdhME3UH4mICA2VQKm4IBmShqz9FQc00%3D&reserved=0>

* Please confirm that all numbers presented in the abstract are present and identical to numbers presented in the main manuscript text.

GENERAL

* In the last sentence of the Abstract Methods and Findings section, please describe the main limitation(s) of the study's methodology.

- We have included the following sentence: A key limitation of this study is the lack of information on patients’ socio-economic profiles.

* In the author summary, in the final bullet point of 'What Do These Findings Mean?', please include the main limitations of the study in non-technical language.

* Ensure all supplementary material has titles and legends, specifically the figures in the supplements.

AUTHOR SUMMARY:

* What did the researchers do and find, 2nd bullet point: Please remove. If you prefer, you may add these numbers in bullet point 1.

* What did the researchers do and find, 3rd bullet point: please change to:

"we found that euthanasia for psychiatric disorders has increased at a similar rate to all euthanasia cases, whereas cases of euthanasia for dementia have increased slightly more."

* What did the researchers do and find: Please consider adding a new bullet point including the information: "there were gender and regional differences in these trends.”

* What do these findings mean, 1st bullet point:  Please rephrase this. I appreciate that the number of euthanasia cases for these conditions is limited, which should be stated here. However, as dementia cases increased more than ‘all’ cases, the statement ‘not led to disproportionate growth’ is not completely accurate.

* What do these findings mean, 2nd bullet point: please consider removing this.

- We have changed the author summary

FUNDING STATEMENT

* Thank you for providing your funding statement. Please also include the URLs for the funders in this statement.

ETHICS AND CONSENT

* Please provide the name(s) of the institutional review board(s) that provided ethical approval.

- We have included this section

OBSERVATIONAL, COHORT, CROSS-SECTIONAL, AND CASE CONTROL STUDIES

* Thank you for including the STROBE checklist in your submission. Please also mention this in the methods section of your manuscript, with the suggested text:  "This study is reported as per the Strengthening the Reporting of Observational Studies in Epidemiology (STROBE) guideline (S1 Checklist)."

- We added the sentence at the end of the methods section.

* Did your study have a prospective protocol or analysis plan? Please state this (either way) early in the Methods section.

a) If a prospective analysis plan (from your funding proposal, IRB or other ethics committee submission, study protocol, or other planning document written before analyzing the data) was used in designing the study, please include the relevant prospectively written document with your revised manuscript as a Supporting Information file to be published alongside your study, and cite it in the Methods section. A legend for this file should be included at the end of your manuscript.

b) If no such document exists, please make sure that the Methods section transparently describes when analyses were planned, and when/why any data-driven changes to analyses took place.

c) In either case, changes in the analysis-- including those made in response to peer review comments-- should be identified as such in the Methods section of the paper, with rationale.

- We did not publish a protocol for this study. We included the following in the methods section: “Original analyses were made using Poisson Regression modelling. We used Zero-Inflated Negative Binomial following reviewers’ recommendations”. (page 7).

Comments from Reviewers:

Reviewer #2:

| **Current Phrase** | **Suggested Alternative** | **Rationale** |  |
| --- | --- | --- | --- |
| “catching up” | “increasing at a faster rate” / “reducing historical differences” | “Catching up” implies progress | We removed the term. |
| “harmonization” | “convergence in rates” | Avoids normative value of “uniformity” | We removed the term. |
| “adjustment across sub-populations” | “shifts in distribution” or “changes across sub-populations” | Neutral alternative | We removed the term. |
| “The Belgian experience addresses this concern.” | “Belgian data offer contextual insights into these concerns.” | Avoids implying resolution of ethical or policy debates | Changed for: “The Belgian experience, however, offers “contextual insights into these concerns.” |
| “not a concern” (in conclusion) | “no observed increase in frequency based on reported data” | Avoids policy-level conclusions | We removed this term. |
| “seems to become more common” | “increased” or “cases have risen over time” | More empirical; avoids conjecture | We did not change this. |
| “differences have narrowed, suggesting harmonization” | “regional differences have declined over time” | Removes implied endorsement | Changed for: “Furthermore, differences across genders and linguistic regions have declined over time.” |

**. Literature and Debate Engagement**

**1. Omission of Qualitative and Clinical Ethical Literature**

The manuscript does not engage with the substantial qualitative research on assisted dying for psychiatric disorders, especially regarding decision-making capacity, physician uncertainty, or patient ambivalence. These perspectives are essential for contextualizing quantitative trends and understanding the ethical implications of ADE expansion.

**Suggestion**: Acknowledge this gap in the limitations section:

“This study focuses on administrative trends and does not address clinical, psychological, or qualitative dimensions of ADE for psychiatric conditions, such as assessments of capacity or decisional ambivalence. These are crucial areas for future research.”

- We have added the following sentence as the end of the first limitation: “Furthermore, this study focuses on administrative trends and does not address clinical, psychological, or qualitative dimensions.”

**2. Underrepresentation of Ethical Diversity**

While the paper attempts to neutralize tone, there is limited engagement with opposing normative frameworks. For example, principled concerns about whether patients with psychiatric suffering can ever make a truly autonomous request for euthanasia are absent, despite their prominence in international debates.

**Suggestion**: In the discussion, include a line such as:

“Our analysis does not resolve ongoing ethical disagreements regarding capacity, suffering, and vulnerability in psychiatric ADE, which remain the subject of principled debate in bioethics and law.”

- As the paper does not include ethical discussions and has a neutral tone, we do not believe such a statement would be necessary.

**3. International Policy Context Still Overstated**

The claim that “the Belgian experience addresses this concern” (re: slippery slope) overstates what the data can prove and implies normative generalizability. Ethical and political contexts differ significantly between Belgium, France, the UK, and elsewhere.

**Suggestion**: Replace with:

“While the Belgian data provide longitudinal insights, caution is warranted in applying these findings to other national contexts where legal frameworks, safeguards, and public attitudes toward assisted dying differ markedly.”

- The point now reads as follow: “The Belgian experience, however, offers contextual insights into these concerns.”

**Final Recommendation**

I commend the authors for the substantial improvements made since R1. This version is very close to being publication ready. With the incorporation of the above adjustments — particularly around normative phrasing, acknowledging ethical diversity, and clarifying limitations — the paper will stand as a rigorous and valuable contribution to the ongoing global debate on assisted dying for psychiatric and cognitive conditions. These are relatively modest changes in wording and framing but important ones, given the ethical sensitivity of the subject matter and the visibility of this paper in future policy discussions. Once these revisions are addressed, I support publication.

- Thank you very much for your final recommendation.
