# Supplementary material for: Trends in assisted dying among patients with psychiatric disorders and dementia in Belgium: A health registry study
Source: PLoS Med. 2025 Nov 19;22(11):e1004522. doi: 10.1371/journal.pmed.1004522 (PMC12646481; doi:10.1371/journal.pmed.1004522)
Supplement: Peer review history

# Editor report: Decision Letter 0

**DOI:** 10.1371/journal.pmed.1004522.r001

**Authors:** Suzanne De Bruijn

7 Jan 2025

Dear Dr Wels,

Thank you for submitting your manuscript entitled "Addressing the Slippery Slope Argument: Trends in Euthanasia Among Patients with Psychiatric Disorders and Dementia in Belgium, 2002–2023" for consideration by PLOS Medicine.

Your manuscript has now been evaluated by the PLOS Medicine editorial staff as well as by an academic editor with relevant expertise and I am writing to let you know that we would like to send your submission out for external peer review.

However, before we can send your manuscript to reviewers, we need you to complete your submission by providing the metadata that is required for full assessment. To this end, please login to Editorial Manager where you will find the paper in the 'Submissions Needing Revisions' folder on your homepage. Please click 'Revise Submission' from the Action Links and complete all additional questions in the submission questionnaire.

Please re-submit your manuscript within two working days, i.e. by Jan 09 2025 11:59PM.

Login to Editorial Manager here: https://www.editorialmanager.com/pmedicine

Once your full submission is complete, your paper will undergo a series of checks in preparation for peer review. Once your manuscript has passed all checks it will be sent out for review.

Feel free to email us at plosmedicine@plos.org if you have any queries relating to your submission.

Kind regards,

Suzanne De Bruijn, PhD

Senior Editor

PLOS Medicine

# Peer review: Decision Letter 1

**DOI:** 10.1371/journal.pmed.1004522.r002

**Authors:** Suzanne De Bruijn

17 Mar 2025

Dear Dr Wels,

Many thanks for submitting your manuscript "Addressing the Slippery Slope Argument: Trends in Euthanasia Among Patients with Psychiatric Disorders and Dementia in Belgium, 2002–2023" (PMEDICINE-D-24-04457R1) to PLOS Medicine. The paper has been reviewed by subject experts and a statistician; their comments are included below and can also be accessed here: [LINK]

As you will see, the reviewers thought this data is valuable, but they also have several major concerns about the study. After discussing the paper with the editorial team and an academic editor with relevant expertise, I'm pleased to invite you to revise the paper in response to the reviewers' comments. In this revision, we would like you to address all reviewer comments. Specifically, we will require the article to be revised to ensure the language is objective in discussing the data. We also ask you to include the relevant literature cited by the reviewers and we would ask you to discuss circumstances that may have influenced euthanasia cases in this time frame, specifically the court case that occurred during the analysed time frame. Although we won't require it, it may also be beneficial to get input from an ethicist. We plan to send the revised paper to some or all of the original reviewers, and we cannot provide any guarantees at this stage regarding publication.

In addition to these revisions, you will need to complete some formatting changes, which you will receive in a follow up email. A member of our team will be in touch with a set of requests shortly.

When you upload your revision, please include a point-by-point response that addresses all of the reviewer and editorial points, indicating the changes made in the manuscript and either an excerpt of the revised text or the location (eg: page and line number) where each change can be found. Please also be sure to check the general editorial comments at the end of this letter and include these in your point-by-point response. When you resubmit your paper, please include a clean version of the paper as the main article file and a version with changes tracked as a marked-up manuscript. It may also be helpful to check the guidelines for revised papers at http://journals.plos.org/plosmedicine/s/revising-your-manuscript for any that apply to your paper.

We ask that you submit your revision by Apr 07 2025 11:59PM. However, I appreciate that the revision we require is substantial, so please feel free to contact me by email to discuss a suitable alternative.

Don't hesitate to contact me directly with any questions (sbruijn@plos.org).

Best regards,

Suzanne

Suzanne De Bruijn, PhD

Associate Editor

PLOS Medicine

sbruijn@plos.org

-----------------------------------------------------------

Comments from the academic editor:

Very interesting set of reviews, all with very legitimate points. I think neutrality of presentation is one clear message coming out of them. The authors should step back and consider their own biases which influence readers, manifest in the language. They should also clearly describe the limitations of this study.

-----------------------------------------------------------

Comments from the reviewers:

Reviewer #1: "Addressing the Slippery Slope Argument: Trends in Euthanasia Among Patients with Psychiatric Disorders and Dementia in Belgium, 2002-2023" analyzed data on all reported cases of euthanasia in Belgium over about two decades via time-series Poisson regression, to determine whether euthanasia trends for psychiatric disorders and dementia indicate a possible "slippery slope". It was found that while euthanasia rates for psychiatric disorders followed similar trends as for other types of euthanasia, dementia had a slight over-increase only partly explained by demographic change. Nevertheless, it was concluded that there was no evidence of significant misuse of euthanasia regulations allowing for non-terminal psychiatric conditions.

While informative, some issues might be considered:

1. Some additional details could be included in the Introduction, to establish the context. In particular,

a) How is euthanasia administered, and what is the typical waiting time between the initial request(s), approval, and administration?

b) Would withdrawal of care count as euthanasia for terminally-ill patients?

c) Is there any specific definition of terminal illness, in terms of expected remaining lifespan and/or suffering?

2. In the Data subsection, it is stated that "The dataset contains aggregated count data stratified by multiple demographic, temporal, and contextual factors. Each row represents a unique combination of attributes, including year, language, gender, age, age group, location, and reason for the case... The dataset is structured as a rectangular table, with each row corresponding to a unique combination of predictor variables, ensuring that all possible combinations are represented, including those with zero counts". This description might be clarified - does it mean that the dataset has 127 unique combinations of (the 7) attributes?

3. In the Reasons for euthanasia subsesction, it might be clarified as to whether the "multimorbidity" category applies whenever a patient would otherwise fall into two or more of the other categories (possibly including psychiatric disorders/dementia).

4. Further, it may be appropriate to consider the baseline prevalence of the various medical conditions, over the time period.

5. In the Population offset subsection, assumptions for language are stated. They might be further justified by relevant citations or otherwise, if possible.

Reviewer #2: See attached file

Reviewer #3: Summary:

This is a population-based register-analysis of aggregated data to investigate the alleged "slippery slope"-phenomena in euthanasia. Data from 2002, the year of initiating euthanasia in Belgium, to 2023 is used and incidence rates and -ratios are calculated to answer questions around the time trend of euthanasia for different underlaying diagnoses, with a particular focus on dementia and psychiatric illnesses. This type of scientific contribution is sorely needed in the field, the aim is admirable, and the analysis are ambitious but there are some clarifications needed, both in analytic approach and in presentation.

Major comments:

1. To me, the text is not objective, but biased in favour of euthanasia, in the choice of word and phrases, please consider revision. (A read-through by a "critical friend" might help)

2. Please clarify the aim in relation to the "slippery slope". Since euthanasia was allowed from the beginning for non-terminal illnesses, including psychiatric ones, in Belgium it is unclear to me what slippery slope that is investigated in the paper. Is it a question of inferring what the more ethically troublesome parts of euthanasia are, and looking into those diagnoses?

3. I acknowledge your take on statistical significance, it is a standpoint but not mine, in my mind Belgium is not the whole world, and 2002-2023 it not all time, therefore, if we wish to generalize the findings to other times and/or places, we need the statistical significance tests. They describe the uncertainty of the data and contribute important information. Especially if one would like to compare IRRs, to say that one is more pronounced than the other, but both estimates are based on very small numbers I think the CIs are very important indeed.

4. I find the analyses difficult to interpret (I note that the authors do not interpret their estimates in words either very much either). Since I am not a statistician, I suggest to the editor for your paper to go to statistical review, but I think there have to be more work done on the analysis, as this is not the customary way to do either IRR or interactions. I would suggest considering a structure like this:

Table 2: Incidence rates (IR) i.e. number of cases/100 000 person-years, for different subtypes of euthanasia, and the total, per year. Be clear in the heading of the table what is shown, the table (with heading and notes) should be self-explanatory.

Table 3: Incidence rate ratios (IRR). Give the estimate by year (e.g. IRR=1.03, there is 3% increase in the probability to die from euthanasia each year). Then, test the statistical significance of the interaction terms that you choose (e.g. There is a statistically significant interaction between year and reason for euthanasia, p=0.03). Then make dummy variables, perhaps grouping the years into categories (e.g. IRR for each year with dementia as reason: 1.02, IRR for each year with other reason: 1.03). (I do not at all require your paper to follow the above exactly, it is intended as a help to describe how an interaction analysis can be presented, I am sure there are many ways, but you current one does not work).

5. What does the first model, that calculate the "occurrence" (frequency?) of euthanasia, contribute with? Does this model take person-time at risk into account at all? Also, I do not find much value in the graphs unfortunately. What does "predicted" mean? Estimated?

6. Interactions are notoriously easy to preform statistically but hard to interpret, please consider excluding the three-way-interactions, (a hint of this is that you yourself state that it is hard to interpret, follow your gut in this case).

Minor comments:

7. Be clear in the year of study start, is it 2002 or 2003?

8. "Informed consent" on p.5 can't possibly be the correct term for requesting euthansia?

9. There are some missing references, e.g. Statbel and R.

10. The incidence rate is called a "prevalence rate" in the method.

11. Twelve medical conditions that are categorized into 7, that are then categorized again into 3: I am not certain that the description of the 7 categories adds value (Methods, p.8).

I reserve the right to add additional comments, primarily on wording, if the authors choose to submit a revised version of the manuscript.

Reviewer #4: This is a scholarly paper in that it uses sophisticated statistical techniques to provide some detailed analysis of complicated data. However, I think you have undermined the academic standing of the paper by your focus (in the paper's title and in its content) on the slippery slope argument. You could publish your results without any reference to the slippery slope argument, in which case the paper would provide something interesting for the research community to ponder. Unfortunately, the focus on the slippery slope argument means that the whole endeavour is wasted.

For a start, if you are going to use your data to attack or undermine the slippery slope argument, you should start with the best account of that argument that you cane find. Instead, you provide only a cursory definition of the argument. Indeed, right from the start (p. 4) you assert that the argument is "fallacious". If the argument is so obviously fallacious, why bother to say anything further. But the argument is not obviously "fallacious". Indeed, in the best hands, there are two aspects to the argument (it's perhaps better to refer to "arguments" in the plural), an empirical aspect and a logical aspect (see Keown (2018) 'Euthanasia, Ethics and Public Policy' pp. 67-89). You might say that you are obviously only applying your analysis to the empirical argument. But that would leave the other aspects of the argument(s) intact, so you would not have achieved your objective and your conclusions would be wrong. In any case, although empirical data can be relevant to the argument, in the end the argument is a normative one which needs to be addressed at a conceptual level not at a statistical one.

There is a very basic point to be made about how the Belgian law demonstrates a slippery slope. The law was passed in 2002 to allow voluntary active euthanasia. Then, in 2014 the Euthanasia Act was amended to allow euthanasia for minors. In so doing, Belgium slid some way down a slippery slope. Full stop. We don't need any further empirical analysis. The eligibility criteria have been extended in keeping with the slippery slope argument(s). But there are lots of other ways in which this slippage can be demonstrated: the move from serious and incurable disorders to multiple disorders, the stretching of the notion of suffering, the inclusion of physician-assisted suicide, and so on. Keown (2018) discusses all of this in some detail (pp.298-325) in a chapter entitled 'Belgium's lack of effective control'. Proper academic discussion of the issue would surely need to include some mention of Keown (2018), where there are two chapter devoted purely to the experience in Belgium. Moreover, I would also have expected some discussion of the book 'Euthanasia and Assisted Suicide: Lessons from Belgium' edited by Jones, Gastmans and MacKellar and published in 2017. One of the things that these works highlight is the number of cases of euthanasia which are not reported, which would also be relevant to a discussion of the possibility of slippery slopes.

There is another point. Whenever euthanasia or assisted suicide are legalised in a country, the rates of "assisted dying" gradually rise. Those against "assisted dying" point to this increasing prevalence as a sign that it is getting out of control; those in favour argue that one would expect the prevalence to increase and suggest it shows that "assisted dying" is acceptable. But, of course, the numbers don't on their own tell us anything, because we don't know what the normal prevalence should be; and, moreover, there is no way of knowing. What is normal here requires an evaluative decision. We need a completely different type of research to say whether or not the prevalence is (normatively) high or low. The same sort of difficulty faces your research. But in any case, the evidence that there is some sort of slippery slope in Belgium is apparent and is not simply based on opinion.

Any attachments provided with reviews can be seen via the following link: [LINK]

--------------------------------------------------------- ---

General editorial requests:

(Note: not all will apply to your paper, but please check each item carefully)

* We ask every co-author listed on the manuscript to fill in a contributing author statement, making sure to declare all competing interests. If any of the co-authors have not filled in the statement, we will remind them to do so when the paper is revised. If all statements are not completed in a timely fashion this could hold up the re-review process. If new competing interests are declared later in the revision process, this may also hold up the submission. Should there be a problem getting one of your co-authors to fill in a statement we will be in contact. Please do not add or remove authors without first discussing this with the handling editor. You can see our competing interests policy here: http://journals.plos.org/plosmedicine/s/competing-interests.

* Please upload any figures associated with your paper as individual TIF or EPS files with 300dpi resolution at resubmission; please read our figure guidelines for more information on our requirements: http://journals.plos.org/plosmedicine/s/figures. While revising your submission, please upload your figure files to the PACE digital diagnostic tool, https://pacev2.apexcovantage.com/. PACE helps ensure that figures meet PLOS requirements. To use PACE, you must first register as a user. Then, login and navigate to the UPLOAD tab, where you will find detailed instructions on how to use the tool. If you encounter any issues or have any questions when using PACE, please email us at PLOSMedicine@plos.org.

* Please ensure that the paper adheres to the PLOS Data Availability Policy (see http://journals.plos.org/plosmedicine/s/data-availability), which requires that all data underlying the study's findings be provided in a repository or as Supporting Information. For data residing with a third party, authors are required to provide instructions with contact information (web or email address) for obtaining the data. Please note that a study author cannot be the contact person for the data. PLOS journals do not allow statements supported by "data not shown" or "unpublished results." For such statements, authors must provide supporting data or cite public sources that include it.

* We expect all researchers with submissions to PLOS in which author-generated code underpins the findings in the manuscript to make all author-generated code available without restrictions upon publication of the work. In cases where code is central to the manuscript, we may require the code to be made available as a condition of publication. Authors are responsible for ensuring that the code is reusable and well documented. Please make any custom code available, either as part of your data deposition or as a supplementary file. Please add a sentence to your data availability statement regarding any code used in the study, e.g. "The code used in the analysis is available from Github [URL] and archived in Zenodo [DOI link]" Please review our guidelines at https://journals.plos.org/plosmedicine/s/materials-software-and-code-sharing and ensure that your code is shared in a way that follows best practice and facilitates reproducibility and reuse. Because Github depositions can be readily changed or deleted, we encourage you to make a permanent DOI'd copy (e.g. in Zenodo) and provide the URL.

FORMATTING - GENERAL

* Abstract: Please structure your abstract using the PLOS Medicine headings (Background, Methods and Findings, Conclusions). Please combine the Methods and Findings sections into one section.

* At this stage, we ask that you include a short, non-technical Author Summary of your research to make findings accessible to a wide audience that includes both scientists and non-scientists. The Author Summary should immediately follow the Abstract in your revised manuscript. This text is subject to editorial change and should be distinct from the scientific abstract. Ideally each sub-heading should contain 2-3 single sentence, concise bullet points containing the most salient points from your study. In the final bullet point of 'What Do These Findings Mean?', please include the main limitations of the study in non-technical language. Please see our author guidelines for more information: https://journals.plos.org/plosmedicine/s/revising-your-manuscript#loc-author-summary.

* Please express the main results with 95% CIs as well as p values. When reporting p values please report as p<0.001 and where higher as the exact p value p=0.002, for example. Throughout, suggest reporting statistical information as follows to improve clarity for the reader "22% (95% CI [13%,28%]; p</=)". Please be sure to define all numerical values at first use.

* Please include page numbers and line numbers in the manuscript file. Use continuous line numbers (do not restart the numbering on each page).

* Please cite the reference numbers in square brackets. Citations should precede punctuation.

FIGURES AND TABLES

* Please provide titles and legends for all figures and tables (including those in Supporting Information files).

* Please define all abbreviations used in each figure/table (including those in Supporting Information files).

* Please consider avoiding the use of red and green in order to make your figure more accessible to those with color blindness.

SUPPLEMENTARY MATERIAL

* Please note that supplementary material will be posted as supplied by the authors. Therefore, please amend it according to the relevant comments outlined here.

* Please cite your Supporting Information as outlined here: https://journals.plos.org/plosmedicine/s/supporting-information

REFERENCES

* PLOS uses the numbered citation (citation-sequence) method and first six authors, et al.

* Please ensure that journal name abbreviations match those found in the National Center for Biotechnology Information (NCBI) databases (http://www.ncbi.nlm.nih.gov/nlmcatalog/journals), and are appropriately formatted and capitalised.

* Where website addresses are cited, please include the complete URL and specify the date of access (e.g. [accessed: 12/06/2023]).

* Please also see https://journals.plos.org/plosmedicine/s/submission-guidelines#loc-references for further details on reference formatting.

OBSERVATIONAL STUDIES

* Abstract: Please include the study design, population and setting, number of participants, years during which the study took place (enrollment and follow up), length of follow up, and main outcome measures.

* Please ensure that the study is reported according to the STROBE (or appropriate STOBE extension) guideline (available from: https://www.equator-network.org/reporting-guidelines/strobe) and include the completed STROBE (or STROBE extension) checklist as Supporting Information. Please add the following statement, or similar, to the Methods: "This study is reported as per the Strengthening the Reporting of Observational Studies in Epidemiology (STROBE) guideline (S1 Checklist)." When completing the checklist, please use section and paragraph numbers, rather than page numbers.

* [FOR POPULATION HEALTH/REGISTRY STUDIES] Please ensure that the study is reported according to the RECORD guideline (available from https://www.record-statement.org) and include the completed checklist as Supporting Information. Please add the following statement, or similar, to the Methods: "This study is reported as per the Reporting of Studies Conducted using Observational Routinely-Collected Data (RECORD) guideline (S1 Checklist)." When completing the checklist, please use section and paragraph numbers, rather than page numbers.

* [FOR POPULATION HEALTH ESTIMATES] Please ensure that the study is reported according to the GATHER statement (available from https://www.equator-network.org/reporting-guidelines/gather-statement) and include the completed checklist as Supporting Information. Please add the following statement, or similar, to the Methods: "This study is reported as per the Guidelines for Accurate and Transparent Health Estimates Reporting (GATHER) statement (S1 Checklist)." When completing the checklist, please use section and paragraph numbers, rather than page numbers.

* For all observational studies, in the manuscript text, please indicate: (1) the specific hypotheses you intended to test, (2) the analytical methods by which you planned to test them, (3) the analyses you actually performed, and (4) when reported analyses differ from those that were planned, transparent explanations for differences that affect the reliability of the study's results. If a reported analysis was performed based on an interesting but unanticipated pattern in the data, please be clear that the analysis was data driven.

* Please state in the Methods section whether the study had a prospective protocol or analysis plan. If a prospective analysis plan (from your funding proposal, IRB or other ethics committee submission, study protocol, or other planning document written before analyzing the data) was used in designing the study, please include the relevant document(s) with your revised manuscript as a Supporting Information file to be published alongside your study and cite it in the Methods section. A legend for this file should be included at the end of your manuscript. If no such document exists, please make sure that the Methods section transparently describes when analyses were planned, and when/why any data-driven changes to analyses took place. Changes in the analysis, including those made in response to peer review comments, should be identified as such in the Methods section of the paper, with rationale.

Attachment Submitted filename: PLOSONE - review.docx

# Author comment: Author response to Decision Letter 2

**DOI:** 10.1371/journal.pmed.1004522.r003

9 May 2025

Attachment Submitted filename: Answers.docx

# Peer review: Decision Letter 2

**DOI:** 10.1371/journal.pmed.1004522.r004

**Authors:** Suzanne De Bruijn

30 Jun 2025

Dear Dr Wels,

Many thanks for submitting your manuscript "Trends in Assisted Dying Among Patients with Psychiatric Disorders and Dementia in Belgium" (PMEDICINE-D-24-04457R2) to PLOS Medicine. The paper has been reviewed by subject experts and a statistician; their comments are included below and can also be accessed here: [LINK]

As you will see, the reviewers thought the manuscript has improved but they still have serious concern about bias. After discussing the paper with the editorial team and an academic editor with relevant expertise, we agreed to offer you one more round of revision to address this issue. We plan to send the revised paper to some or all of the original reviewers, and we cannot provide any guarantees at this stage regarding publication.

Specifically we would like you to address the following:

1) Revise the language sentence by sentence. This applies to the whole manuscript, but the Abstract and Discussion in particular.

Specific points:

-remove the causal language on line 36 of the abstract.

-remove line 42-44 of the abstract and re-write this.

2) Please remove any use of the slippery slope argument completely, and instead just focus on the examination of the trends in euthanasia cases. This can be along the lines of “Legislation for ADE was adopted in 2002 in Belgium permitting euthanasia for terminal illness and for non-terminal illnesses such as XYZ. In this study we examined trends in euthanasia cases involving patients psychiatric disorders, dementia and terminal illness from 200X-20XX”

3) Please remove the court case discussion in the introduction, instead it would be fine to state it’s a contentions issue. Court cases could be mentioned in the discussion only.

4) Please shorten your introduction to 750 words.

5) Please trim the discussion, as this is overly long currently.

In addition to these revisions, you will need to complete some formatting changes, which you will receive in a follow up email. A member of our team will be in touch with a set of requests shortly.

When you upload your revision, please include a point-by-point response that addresses all of the reviewer and editorial points, indicating the changes made in the manuscript and either an excerpt of the revised text or the location (eg: page and line number) where each change can be found. Please also be sure to check the general editorial comments at the end of this letter and include these in your point-by-point response. When you resubmit your paper, please include a clean version of the paper as the main article file and a version with changes tracked as a marked-up manuscript. It may also be helpful to check the guidelines for revised papers at http://journals.plos.org/plosmedicine/s/revising-your-manuscript for any that apply to your paper.

We ask that you submit your revision by Jul 21 2025 11:59PM. However, if this deadline is not feasible, please contact me by email, and we can discuss a suitable alternative.

Don't hesitate to contact me directly with any questions (sbruijn@plos.org).

Best regards,

Suzanne

Suzanne De Bruijn, PhD

Associate Editor

PLOS Medicine

sbruijn@plos.org

-----------------------------------------------------------

Comments from the academic editor:

Please ensure that they text is neutral. I suggest to examine every sentence to consider whether the text is justified, and whether there is any implicit bias still there. Maybe imagine you are someone very much against assisted suicide, and read the manuscript from their perspective.

-----------------------------------------------------------

Comments from the reviewers:

Reviewer #1: We thank the authors for addressing our previous comments, and the other reviewers for improving the statistical analyses.

Reviewer #2: I sincerely thank the authors for carefully addressing the reviewers' comments. The revisions have significantly improved the clarity and quality of the manuscript.

There still remains minor issues to be addressed:

1. Terminology: Euthanasia vs. Assisted Suicide

The clarification provided in the methods and background sections regarding the exclusive use of "euthanasia" in line with Belgian legal definitions is well explained. However, I recommend adding a brief footnote at the first mention of the term "euthanasia" to inform international readers that in Belgium, this may include rare cases of supervised self-administration that are still legally classified as euthanasia.

Concerning terminologia, i point that assisted dying is commonly used as an umbrella term , including euthanasia and assisted suicide. For this reason, I suggests that authors to use only the term assisted dying and not assisted dying and euthanasia.

2. Interpretation of the Slippery Slope Argument

The revised manuscript commendably distinguishes between the empirical and normative dimensions of the "slippery slope" argument.

Suggestion:

I recommend including an explicit statement in the discussion acknowledging that while the empirical data do not support a slippery slope in numerical terms, ethical concerns about the broadening of eligibility criteria remain valid and deserve further comparative research.

3. Gender and Regional Trends

The authors have appropriately acknowledged data limitations and refrained from speculation regarding gender and regional disparities.

Further Recommendation:

Consider moving part of this discussion into the limitations section, where it may be more visible.

4. Policy and Limitations

The discussion section has been usefully restructured.

Additional Suggestion:

The authors may wish to state more explicitly that while dementia-related euthanasia remains infrequent, the upward trend over time is noteworthy and warrants monitoring.

5. Minor issues

- The shift to a zero-inflated negative binomial model is appropriate and well justified.

- Including 95% confidence intervals greatly improves transparency.

- Some defensive phrasing remains, especially regarding critical literature (e.g., references to the 2017 edited volume). I encourage the authors to maintain a more neutral and academic tone throughout.

Conclusion

Overall, this is a strong and timely study. With a few remaining refinements to language and emphasis, I believe the manuscript will be ready for publication.

Reviewer #3: I acknowledge that the authors have invested into the revision of the manuscript, and improvements has been achieved, however there is still significant issues remaining.

1. Formulations in the text still puts into question the neutrality of the authors in terms of euthanasia as a concept. Would one for example use the phrase "catching up" if cancer incidence of one region were approaching another region with higher incidence? This is not easily fixed with further revisions, since the text is a sign of the problem, rather than the problem itself.

Also, in abstract conclusion is stated "…without evidence of significant misuse…" the misuse, or lack of it, has not been investigated, only time trends. The fact that this is the conclusion after another reviewer pointed out that the conclusions were overreaching is troublesome.

2. There is a lack of scientific integrity shown in the discrepancy between the answer to me regarding 95%CIs: "We have now added 95% confidence intervals (CIs) to the paper, as we agree they contribute valuable information for interpreting the robustness of the results." Vs the text in the manuscript: "the 95% CIs are unnecessary and do not provide meaningful insights." (row 412 in tracked changes version).

In addition, the authors state that they do not wish to extrapolate the findings outside of Belgium. In the second paragraph of the Discussion (rows 569-584) the authors reason around the situation in UK and France, concluding "The Belgian experience, however, addresses this concern." I don't mind this, I fact I think this is why we do research at all: to be able to extrapolate into the unknown, (the etiology of cancer tumours not yet developed e.g.), but the fact the authors don't see that this is what they doing is concerning.

3. I appreciate the decreased focus and usage of the term/concept "slippery slope", however, it is still present (e.g. in the conclusion of the Abstract). This is from the revised abstract: "Assisted dying and euthanasia (ADE) for patients with psychiatric disorders or dementia have increased in countries where the practice is legal. These cases are often cited in arguments against ADE legislation, as they are perceived to contribute to a potential slippery slope in case numbers." The fact that the case numbers increase, or not, does not tell us if that is good (good uptake of new legislation, the opportunity of new treatment reaches all part of the population) or bad (misuse, lax definition of eligible patients), that is an ethical interpretation, not empirical. It is built into the "slippery slope"-argument that an increase is bad, but the increase itself does not carry any value when not coupled with an ethical standpoint. Which is why I would wish the authors really kept only to analyses of the data and conclusion that can be drawn from these.

4. In my first review I asked "What does the first model, that calculate the "occurrence" (frequency?) of euthanasia, contribute with?" this was not answered but the manuscript now states "Importantly, this model treats the outcome purely as a count, without accounting for differences in population size." This would seem to indicate that Model 1 adds no value to the manuscript and should be removed. (Or, an explanation in the manuscript of how the interpretation of model 1 helps the final conclusion is needed)

(This remind me of papers of my own where we started analysis somewhere, moved on in our thinking and did not until late realize that the first attempts were superfluous in the manuscript, even if they were instrumental for our moving forwards in analytical stage)

Any attachments provided with reviews can be seen via the following link: [LINK]

--------------------------------------------------------- ---

General editorial requests:

* Please ensure that the study is reported according to the Strobe guideline and include the completed Strobe checklist as Supporting Information. When completing the checklist, please use section and paragraph numbers, rather than page numbers. Please add the following statement, or similar, to the Methods: "This study is reported as per Strobe guideline (S1 Checklist)."

* For all observational studies, in the manuscript text, please indicate: (1) the specific hypotheses you intended to test, (2) the analytical methods by which you planned to test them, (3) the analyses you actually performed, and (4) when reported analyses differ from those that were planned, transparent explanations for differences that affect the reliability of the study's results. If a reported analysis was performed based on an interesting but unanticipated pattern in the data, please be clear that the analysis was data driven.

* Please state in the Methods section whether the study had a prospective protocol or analysis plan. If a prospective analysis plan (from your funding proposal, IRB or other ethics committee submission, study protocol, or other planning document written before analyzing the data) was used in designing the study, please include the relevant document(s) with your revised manuscript as a Supporting Information file to be published alongside your study and cite it in the Methods section. A legend for this file should be included at the end of your manuscript. If no such document exists, please make sure that the Methods section transparently describes when analyses were planned, and when/why any data-driven changes to analyses took place. Changes in the analysis, including those made in response to peer review comments, should be identified as such in the Methods section of the paper, with rationale.

# Author comment: Author response to Decision Letter 3

**DOI:** 10.1371/journal.pmed.1004522.r005

31 Aug 2025

Attachment Submitted filename: Reviews-2.docx

# Peer review: Decision Letter 3

**DOI:** 10.1371/journal.pmed.1004522.r006

**Authors:** Suzanne De Bruijn

8 Oct 2025

Dear Dr. Wels,

Thank you very much for re-submitting your manuscript "Trends in Assisted Dying Among Patients with Psychiatric Disorders and Dementia in Belgium" (PMEDICINE-D-24-04457R3) for review by PLOS Medicine.

I have discussed the paper with my colleagues and the academic editor and it was also seen again by 1 reviewer. I am pleased to say that provided the remaining editorial and production issues are dealt with we are planning to accept the paper for publication in the journal. However, please note that we will require you to incorporate the textual suggestions from reviewer #2, as well as the textual suggestions from the editors. Furthermore, there are several editorial issues that need to be addressed, which are listed at the end of this email.

Any accompanying reviewer attachments can be seen via the link below. Please take these into account before resubmitting your manuscript:

[LINK]

***Please note while forming your response, if your article is accepted, you may have the opportunity to make the peer review history publicly available. The record will include editor decision letters (with reviews) and your responses to reviewer comments. If eligible, we will contact you to opt in or out.***

In revising the manuscript for further consideration here, please ensure you address the specific points made by each reviewer and the editors. In your rebuttal letter you should indicate your response to the reviewers' and editors' comments and the changes you have made in the manuscript. Please submit a clean version of the paper as the main article file. A version with changes marked must also be uploaded as a marked up manuscript file.

Please also check the guidelines for revised papers at http://journals.plos.org/plosmedicine/s/revising-your-manuscript for any that apply to your paper. If you haven't already, we ask that you provide a short, non-technical Author Summary of your research to make findings accessible to a wide audience that includes both scientists and non-scientists. The Author Summary should immediately follow the Abstract in your revised manuscript. This text is subject to editorial change and should be distinct from the scientific abstract.

In addition to these revisions, you may need to complete some formatting changes, which you will receive in a follow up email. A member of our team will be in touch with a set of requests shortly. If you do not receive a separate email within a few days, please assume that checks have been completed, and no additional changes are required.

We expect to receive your revised manuscript within 1 week. Please email us (plosmedicine@plos.org) if you have any questions or concerns.

We ask every co-author listed on the manuscript to fill in a contributing author statement. If any of the co-authors have not filled in the statement, we will remind them to do so when the paper is revised. If all statements are not completed in a timely fashion this could hold up the re-review process. Should there be a problem getting one of your co-authors to fill in a statement we will be in contact. YOU MUST NOT ADD OR REMOVE AUTHORS UNLESS YOU HAVE ALERTED THE EDITOR HANDLING THE MANUSCRIPT TO THE CHANGE AND THEY SPECIFICALLY HAVE AGREED TO IT.

Please ensure that the paper adheres to the PLOS Data Availability Policy (see http://journals.plos.org/plosmedicine/s/data-availability), which requires that all data underlying the study's findings be provided in a repository or as Supporting Information. For data residing with a third party, authors are required to provide instructions with contact information for obtaining the data. PLOS journals do not allow statements supported by "data not shown" or "unpublished results." For such statements, authors must provide supporting data or cite public sources that include it.

To enhance the reproducibility of your results, we recommend that you deposit your laboratory protocols in protocols.io, where a protocol can be assigned its own identifier (DOI) such that it can be cited independently in the future. Additionally, PLOS ONE offers an option to publish peer-reviewed clinical study protocols. Read more information on sharing protocols at https://plos.org/protocols?utm_medium=editorial-email&utm_source=authorletters&utm_campaign=protocols

Please review your reference list to ensure that it is complete and correct. If you have cited papers that have been retracted, please include the rationale for doing so in the manuscript text, or remove these references and replace them with relevant current references. Any changes to the reference list should be mentioned in the rebuttal letter that accompanies your revised manuscript.

Please note, when your manuscript is accepted, an uncorrected proof of your manuscript will be published online ahead of the final version, unless you've already opted out via the online submission form. If, for any reason, you do not want an earlier version of your manuscript published online or are unsure if you have already indicated as such, please let the journal staff know immediately at plosmedicine@plos.org.

If you have any questions in the meantime, please contact me or the journal staff on plosmedicine@plos.org.  

We look forward to receiving the revised manuscript by Oct 22 2025 11:59PM.   

Sincerely,

Suzanne De Bruijn, PhD

Associate Editor 

PLOS Medicine

plosmedicine.org

------------------------------------------------------------

Requests from Editors:

* Please use the active voice throughout the manuscript.

* Please remove any use of the phrase 'uncontrolled increase'

* Please move the ‘limitations’ section to the discussion.

Specific textual requests:

ABSTRACT

Line 20-24 (background): Please change to:

“Assisted dying and euthanasia (ADE) laws make a distinction between ADE for terminal illnesses and non-terminal illnesses such as psychiatric disorders or dementia. ADE for patients with psychiatric disorders or dementia have increased in countries where the practice is legal. In this study, we examine trends in euthanasia cases involving patients with these conditions in Belgium, between 2002 and 2023.

Line 32-35 (methods and findings). Please change to:

“Although slightly increasing, euthanasia for psychiatric disorders followed trends similar to the other types of euthanasia (count=1.00 [95%CI: 0.98; 1.03] – rate=1.02 [95%CI: 0.99; 1.04]), while euthanasia cases for dementia increased faster than other types of dementia (count= 1.03 [95%CI: 1.00; 1.06] – rate= 1.04 [95%CI: 36 1.01;1.07]).

Line 41: please change ‘discussion’ into ‘Conclusion’.

Line 41- 46 (Conclusion): Please change to:

“In Belgium, between 2002 and 2023, there are distinct trends for euthanasia for non-terminal illnesses. Euthanasia for psychiatric disorders followed similar trends as euthanasia for terminal illnesses, whereas euthanasia cases involving cognitive conditions increased at a faster rate. Furthermore, there were gender and regional differences, which diminished over time.”

BACKGROUND

Line 58-60: change to:

“The discourse primarily focuses on two categories of conditions where death is not expected within 12 months: dementia and other degenerative disorders, and severe psychiatric illnesses.”

Line 65-67: remove ‘actual’ , remove ‘leaving little room for empirical evidence’.

Line 67: remove ‘Similarly’.

Line 71: change ‘demand’ to ‘numbers of cases’

Line 73-76: change to:

“The observed increase in cases fuel concerns about a gradual expansion of eligibility criteria, particularly leading to concerns regarding vulnerable patients. These concerns are used as an argument against implementing ADE for non-terminal illness in other jurisdictions and as an argument in favour of introducing stricter safeguards.”

Line 80-82:change to:

“the idea that eligibility criteria in ADE regulations are gradually expanded, or that vulnerable populations are at greater risk is not substantiated by empirical studies.”

Line 83 remove ‘notably’ before focused.

RESULTS:

Line 258-261: Please change to:

“Over the selected period, psychiatric disorders and dementia represent 1.27% and 0.92% of all cases, respectively, and followed similar trends to the overall number of cases.”

Line 264: Besides psychiatric conditions and cognitive disorders, are there any other non-terminal illnesses reason for euthanasia? If not, please change ‘with those for other reasons’ to ‘with those for terminal illnesses’.

Line 288-293: Change to:

“the difference between predicted rates with and without adjustment for population composition and change suggests that population change may partially underlie changes observed in euthanasia prevalence. Furthermore, we observe that the rate of increase in euthanasia cases is similar for terminal illnesses and psychiatric disorders, but higher for dementia cases.”

Line 311-313: Change to:

‘However, over time the rate of increase is becoming larger in Wallonia-Brussels compared with Flanders, indicating that the observed difference between these two regions is diminishing.’

Line 322-324: what is meant here? ‘other’ reasons involving mental suffering? Or all euthanasia cases for ‘other’ (which sounds unlikely?). Please clarify.

Line 328-329: Please remove this sentence, as there is no statistical significance for these findings.

Line 341-347: change to:

“These cases have been more prevalent in the Dutch-speaking community than the French-speaking community, although this regional difference has diminished over time. Furthermore, we observed that most of the increase in psychiatric and dementia-related euthanasia occurred in the French-speaking community.”

Line 350-353: Please remove this paragraph. Especially the last sentence ‘Overall, much of the increase in euthanasia cases is driven by short-term death expectations, rather than long-term ones.’ as this statement is not directly linked to the goal of the study.

Line 360-363: Please remove these 2 sentences, as it seems overly political, and can be perceived as bias.

Line 367: remove ‘proactively’

Line 370-375: Please reconsider this paragraph. ‘methods used to analyse ADE figures….’ As this seems to criticize other studies, rather than highlighting a limitation of the current study.

DISCUSSION:

Line 385-396: change to:

“Recent debates on the implementation of assisted dying in the united Kingdom and France have highlighted the distinction between euthanasia for terminal- and non-terminal causes, with both these countries considering implementation of euthanasia for terminal-illnesses only, excluding patients with psychiatric disorders or degenerative conditions.

In contrast, Belgium euthanasia law has allowed euthanasia for non-terminal and non-physical illnesses from its inception in 2002, allowing us to compare rates in euthanasia for terminal illnesses, psychiatric disorders, and dementia.

Line 397-: remove ‘dramatic increase’. Suggested text:

“In 2023, psychiatric disorders and dementia accounted for 1.4% and 1.2% of all cases, respectively. We observed a significant, but still modest, increase in euthanasia for dementia. Furthermore, we observed that differences across genders and linguistic regions have narrowed over time.

Line 414-418: Please consider removing this paragraph; without data on changes in quantity/quality of palliative care units, this adds little to the discussion.

Line 419-435; please change the last paragraph to:

“Concerns have been raised, both in parliamentary debates [39] and within professional medical bodies, that permitting euthanasia in limited cases (e.g., terminal illness) may gradually lead to less justified applications, and whether safeguards are effectively maintained when euthanasia is extended to non-terminal conditions or psychiatric disorders [41] . Whilst we acknowledge that empirical data cannot resolve ethical and normative questions, our findings demonstrate that euthanasia for psychiatric and cognitive conditions, which has been legally permissible in Belgium since 2002, has not substantially increased between 2002 and 2023. These findings can add to the discussion of end-of-life issues and their inherent complexities.”

GENERAL EDITORIAL REQUESTS

* Thank you for providing an Author summary. Can you please include this within the main manuscript, directly following the Abstract. In the final bullet point of ‘What Do These Findings Mean?’ Please include the main limitations of the study in non-technical language.

* Please confirm that your title complies with to PLOS Medicine's style. Your title must be nondeclarative and not a question. It should begin with main concept if possible. "Effect of" should be used only if causality can be inferred, i.e., for an RCT. Please place the study design ("A randomized controlled trial," "A retrospective study," "A modelling study," etc.) in the subtitle (ie, after a colon).

* Please confirm that your abstract complies with our requirements, including format (three sections: Background, Methods and Findings, and Conclusions) and providing all the information relevant to this study type https://journals.plos.org/plosmedicine/s/submission-guidelines#loc-abstract

* Please confirm that all numbers presented in the abstract are present and identical to numbers presented in the main manuscript text.

GENERAL

* In the last sentence of the Abstract Methods and Findings section, please describe the main limitation(s) of the study's methodology.

* In the author summary, in the final bullet point of 'What Do These Findings Mean?', please include the main limitations of the study in non-technical language.

* Ensure all supplementary material has titles and legends, specifically the figures in the supplements.

AUTHOR SUMMARY:

* What did the researchers do and find, 2nd bullet point: Please remove. If you prefer, you may add these numbers in bullet point 1.

* What did the researchers do and find, 3rd bullet point: please change to:

"we found that euthanasia for psychiatric disorders has increased at a similar rate to all euthanasia cases, whereas cases of euthanasia for dementia have increased slightly more."

* What did the researchers do and find: Please consider adding a new bullet point including the information: "there were gender and regional differences in these trends.”

* What do these findings mean, 1st bullet point: Please rephrase this. I appreciate that the number of euthanasia cases for these conditions is limited, which should be stated here. However, as dementia cases increased more than ‘all’ cases, the statement ‘not led to disproportionate growth’ is not completely accurate.

* What do these findings mean, 2nd bullet point: please consider removing this.

FUNDING STATEMENT

* Thank you for providing your funding statement. Please also include the URLs for the funders in this statement.

ETHICS AND CONSENT

* Please provide the name(s) of the institutional review board(s) that provided ethical approval.

OBSERVATIONAL, COHORT, CROSS-SECTIONAL, AND CASE CONTROL STUDIES

* Thank you for including the STROBE checklist in your submission. Please also mention this in the methods section of your manuscript, with the suggested text: "This study is reported as per the Strengthening the Reporting of Observational Studies in Epidemiology (STROBE) guideline (S1 Checklist)."

* Did your study have a prospective protocol or analysis plan? Please state this (either way) early in the Methods section.

a) If a prospective analysis plan (from your funding proposal, IRB or other ethics committee submission, study protocol, or other planning document written before analyzing the data) was used in designing the study, please include the relevant prospectively written document with your revised manuscript as a Supporting Information file to be published alongside your study, and cite it in the Methods section. A legend for this file should be included at the end of your manuscript.

b) If no such document exists, please make sure that the Methods section transparently describes when analyses were planned, and when/why any data-driven changes to analyses took place.

c) In either case, changes in the analysis-- including those made in response to peer review comments-- should be identified as such in the Methods section of the paper, with rationale.

Comments from Reviewers:

Reviewer #2:

see attached document

Any attachments provided with reviews can be seen via the following link:

[LINK]

Attachment Submitted filename: Review 3. PLOS one. 2025.docx

# Author comment: Author response to Decision Letter 4

**DOI:** 10.1371/journal.pmed.1004522.r007

9 Oct 2025

Attachment Submitted filename: Response to reviewers.docx

# Editor report: Decision Letter 4

**DOI:** 10.1371/journal.pmed.1004522.r008

**Authors:** Suzanne De Bruijn

16 Oct 2025

Dear Dr. Wels,

Thank you very much for re-submitting your manuscript "Trends in Assisted Dying Among Patients with Psychiatric Disorders and Dementia in Belgium" (PMEDICINE-D-24-04457R4) for review by PLOS Medicine.

However we have several remaining editorial requests that we would like you to address.

* Please change your title to: "Trends in Assisted Dying Among Patients with Psychiatric Disorders and Dementia in Belgium: a health registry study"

* I noted the limitations are copied to the discussion as requested, but not removed from their original location. Please remove this to ensure the text is not duplicated.

* Author summary: Please move the limitations from 'what did the researchers do and find' to 'what do these findings mean?"

* FUNDING STATEMENT:

Thank you for providing your funding statement. Please also include the URLs for the funders in this statement

* ETHICS STATEMENT: Please also provide an IRB approval number.

* Line 264: could you give a bit more detail about multi-morbidity, given that it's a non-terminal area not covered by the analysis.

* Line 316: there is a typo: please change 'in' to 'is'.

* Line 412: Please edit this paragraph to: "During the first five years following the implementation of the regulation, cases of euthanasia for psychiatric disorders and dementia remained infrequent. Differences in access across genders and linguistic regions have narrowed over time".

Further there were 2 additional comments from the Academic Editor:

-lines 336-368 and 346-348: the units and the way these are representated are likely to be opaque for readers; is there a way to make them more meaningful to interpret? one possibility is events/population/time unit, and not using so many decimal points. This would be simply for clarity.

-Agree with your rebuttal on the comment regarding line 328-329.

***Please note while forming your response, if your article is accepted, you may have the opportunity to make the peer review history publicly available. The record will include editor decision letters (with reviews) and your responses to reviewer comments. If eligible, we will contact you to opt in or out.***

In revising the manuscript for further consideration here, please ensure you address the specific points made by each reviewer and the editors. In your rebuttal letter you should indicate your response to the reviewers' and editors' comments and the changes you have made in the manuscript. Please submit a clean version of the paper as the main article file. A version with changes marked must also be uploaded as a marked up manuscript file.

Please also check the guidelines for revised papers at http://journals.plos.org/plosmedicine/s/revising-your-manuscript for any that apply to your paper. If you haven't already, we ask that you provide a short, non-technical Author Summary of your research to make findings accessible to a wide audience that includes both scientists and non-scientists. The Author Summary should immediately follow the Abstract in your revised manuscript. This text is subject to editorial change and should be distinct from the scientific abstract.

In addition to these revisions, you may need to complete some formatting changes, which you will receive in a follow up email. A member of our team will be in touch with a set of requests shortly. If you do not receive a separate email within a few days, please assume that checks have been completed, and no additional changes are required.

We expect to receive your revised manuscript within 1 day. Please email us (plosmedicine@plos.org) if you have any questions or concerns.

Please note, when your manuscript is accepted, an uncorrected proof of your manuscript will be published online ahead of the final version, unless you've already opted out via the online submission form. If, for any reason, you do not want an earlier version of your manuscript published online or are unsure if you have already indicated as such, please let the journal staff know immediately at plosmedicine@plos.org.

If you have any questions in the meantime, please contact me or the journal staff on plosmedicine@plos.org.  

We look forward to receiving the revised manuscript by Oct 18 2025 11:59PM.   

Sincerely,

Suzanne De Bruijn, PhD

Associate Editor 

PLOS Medicine

plosmedicine.org

# Editor report: Decision Letter 5

**DOI:** 10.1371/journal.pmed.1004522.r009

**Authors:** Suzanne De Bruijn

29 Oct 2025

Dear Dr Wels, 

On behalf of my colleagues and the Academic Editor, Carol Brayne, I am pleased to inform you that we have agreed to publish your manuscript "Trends in Assisted Dying Among Patients with Psychiatric Disorders and Dementia in Belgium: a health registry study" (PMEDICINE-D-24-04457R5) in PLOS Medicine.

As a final request, we would ask you to include some more detail into your ethics statement, regarding how approval from the FCCEE was obtained.

Furthermore, before your manuscript can be formally accepted you will need to complete some formatting changes, which you will receive in a follow up email. Please be aware that it may take several days for you to receive this email; during this time no action is required by you. Once you have received these formatting requests, please note that your manuscript will not be scheduled for publication until you have made the required changes.

In the meantime, please log into Editorial Manager at http://www.editorialmanager.com/pmedicine/, click the "Update My Information" link at the top of the page, and update your user information to ensure an efficient production process. 

PRESS

We frequently collaborate with press offices. If your institution or institutions have a press office, please notify them about your upcoming paper at this point, to enable them to help maximise its impact. If the press office is planning to promote your findings, we would be grateful if they could coordinate with medicinepress@plos.org. If you have not yet opted out of the early version process, we ask that you notify us immediately of any press plans so that we may do so on your behalf.

We also ask that you take this opportunity to read our Embargo Policy regarding the discussion, promotion and media coverage of work that is yet to be published by PLOS. As your manuscript is not yet published, it is bound by the conditions of our Embargo Policy. Please be aware that this policy is in place both to ensure that any press coverage of your article is fully substantiated and to provide a direct link between such coverage and the published work. For full details of our Embargo Policy, please visit http://www.plos.org/about/media-inquiries/embargo-policy/.

Thank you again for submitting to PLOS Medicine. We look forward to publishing your paper. 

Sincerely, 

Suzanne De Bruijn, PhD 

Associate Editor 

PLOS Medicine
